# scMAGIC: accurately annotating single cells using two rounds of reference-based classification

**DOI:** 10.1093/nar/gkab1275

**Published:** 2022-01-05

**Authors:** Yu Zhang, Feng Zhang, Zekun Wang, Siyi Wu, Weidong Tian

**Affiliations:** State Key Laboratory of Genetic Engineering, Collaborative Innovation Center for Genetics and Development, Department of Computational Biology, School of Life Sciences, Fudan University, Shanghai 200438, P.R. China; State Key Laboratory of Genetic Engineering, Collaborative Innovation Center for Genetics and Development, Department of Computational Biology, School of Life Sciences, Fudan University, Shanghai 200438, P.R. China; Department of Histoembryology, Genetics and Developmental Biology, Shanghai Key Laboratory of Reproductive Medicine, Key Laboratory of Cell Differentiation and Apoptosis of Chinese Ministry of Education, Shanghai Jiao Tong University School of Medicine, Shanghai 200025, China; State Key Laboratory of Genetic Engineering, Collaborative Innovation Center for Genetics and Development, Department of Computational Biology, School of Life Sciences, Fudan University, Shanghai 200438, P.R. China; State Key Laboratory of Genetic Engineering, Collaborative Innovation Center for Genetics and Development, Department of Computational Biology, School of Life Sciences, Fudan University, Shanghai 200438, P.R. China; State Key Laboratory of Genetic Engineering, Collaborative Innovation Center for Genetics and Development, Department of Computational Biology, School of Life Sciences, Fudan University, Shanghai 200438, P.R. China; Qilu Children's Hospital of Shandong University, No 23976 Jingshi Road, Jinan, Shandong, China; Children’s Hospital of Fudan University, Shanghai 201102, China

## Abstract

Here, we introduce scMAGIC (**S**ingle **C**ell annotation using **MA**rker **G**enes **I**dentification and two rounds of reference-based **C**lassification [RBC]), a novel method that uses well-annotated single-cell RNA sequencing (scRNA-seq) data as the reference to assist in the classification of query scRNA-seq data. A key innovation in scMAGIC is the introduction of a second-round RBC in which those query cells whose cell identities are confidently validated in the first round are used as a new reference to again classify query cells, therefore eliminating the batch effects between the reference and the query data. scMAGIC significantly outperforms 13 competing RBC methods with their optimal parameter settings across 86 benchmark tests, especially when the cell types in the query dataset are not completely covered by the reference dataset and when there exist significant batch effects between the reference and the query datasets. Moreover, when no reference dataset is available, scMAGIC can annotate query cells with reasonably high accuracy by using an atlas dataset as the reference.

## INTRODUCTION

Single-cell RNA sequencing (scRNA-seq) technology has been widely used for investigating heterogeneity in tissues and tumors, leading to a better understanding on development and tumorigenesis (1,2). A critical step in scRNA-seq data analysis is the classification of single cells, which is typically done by first clustering cells according to their expression profiles and then annotating each cell cluster using known cell type-specific marker genes (3). However, in order to select appropriate marker gene sets, it is often unavoidable to conduct extensive literature review and manually test various combination of marker genes, which is not only time-consuming but also error-prone. It is therefore highly desirable to develop an automated method that can accurately classify single cells without manual selection of marker genes.

There are now a wealth of scRNA-seq data produced from different tissues, such as brain (4), pancreas (5), peripheral blood mononuclear cells (PBMC) (6) etc. with many well annotated at the level of cell types. Recently, several large-scale scRNA-seq studies have constructed the atlas of single-cells, such as the Human Cell Landscape (HCL) (7) and the Mouse Cell Atlas (MCA) (8), providing the annotations for hundreds of cell types. A number of reference-based classification (RBC) methods have thus been developed to use the expression profiles of known cell types as the reference to assist in the classification of query cells (9–21). These RBC methods generally first identify the characteristic features for each reference cell type, such as the marker genes, and then use these features to assess the similarity of a query cell with each reference cell type, from which the most relevant one is selected to label the query cell. Depending on the way of similarity evaluation, these RBC methods can be classified into four categories: statistical-metric-based methods [e.g. SingleR (9), scmap (10) and SciBet (11)], tree-based methods [e.g. CHETAH (12) and scClassify (13)], machine-learning-based methods [e.g. scPred (14), SingleCellNet (15), scID (16), CaSTLe (17) and SVM_rejection_ (18)] and semi-supervised-learning-based methods [e.g. Seurat v4 (19), scSemiCluster (20) and CALLR (21)]. The first three categories of methods directly compare the query expression profiles against the reference expression profiles and are therefore prone to be affected by the batch effects between the reference and the query datasets. The semi-supervised-learning-based methods are more resistant to the batch effects in that they essentially integrate the reference and query cells’ expression profiles into the same space before doing the classification, during which the batch effects are reduced. However, the batch effects may still affect the quality of the integration and consequently the performance of the classification. Besides the batch effects, the coverage of query cell types by the reference dataset may also affect the performance of these methods. In general, all RBC methods work well when the cell types in the query dataset are all covered by the reference dataset. However, when the query dataset includes cells whose cell types are not covered by the reference dataset, i.e. unclassifiable query cells, the cell type-specific features identified by these RBC methods may not be specific and discriminative enough to distinguish unclassifiable query cells from classifiable ones.

Here, we present scMAGIC (**S**ingle **C**ell annotation using **MA**rker **G**enes **I**dentification and two rounds of reference-based **C**lassification), a novel RBC tool that provides solutions to the above mentioned challenges. The main idea of scMAGIC is to first identify a candidate reference cell type for a query cell by expression profile correlation and then validate its cell identity using the marker genes of the reference cell type. Different from other RBC methods, scMAGIC identifies the marker genes of a reference cell type based not only on the reference dataset but also on an additional atlas dataset that consists of the expression profiles of hundreds of cell types, ensuring that the marker genes are more biologically relevant to the reference cell type. In addition, scMAGIC introduces a second-round RBC in which those query cells whose cell identifies are confidently validated in the first-round RBC are used as the new reference to again classify query single cells, eliminating the batch effects between the reference and the query dataset and therefore significantly improving the classification results. We have designed three situations that users may encounter when conducting RBC and have prepared a total of 86 benchmark tests using real datasets for these situations. Compared with 13 recently developed RBC methods, scMAGIC consistently achieves superior performance in all three situations, and its advantage over other methods is particularly significant when the cell types in the query dataset are not completely covered by the reference dataset and when there exist significant batch effects in between the reference and query datasets. In addition, when no specific reference dataset is available, scMAGIC can still annotate query cells with reasonably high accuracy by using an atlas dataset as the reference. scMAGIC is implemented in R and is available at https://github.com/TianLab-Bioinfo/scMAGIC.

## MATERIALS AND METHODS

### Input for scMAGIC

**Figure 1.**
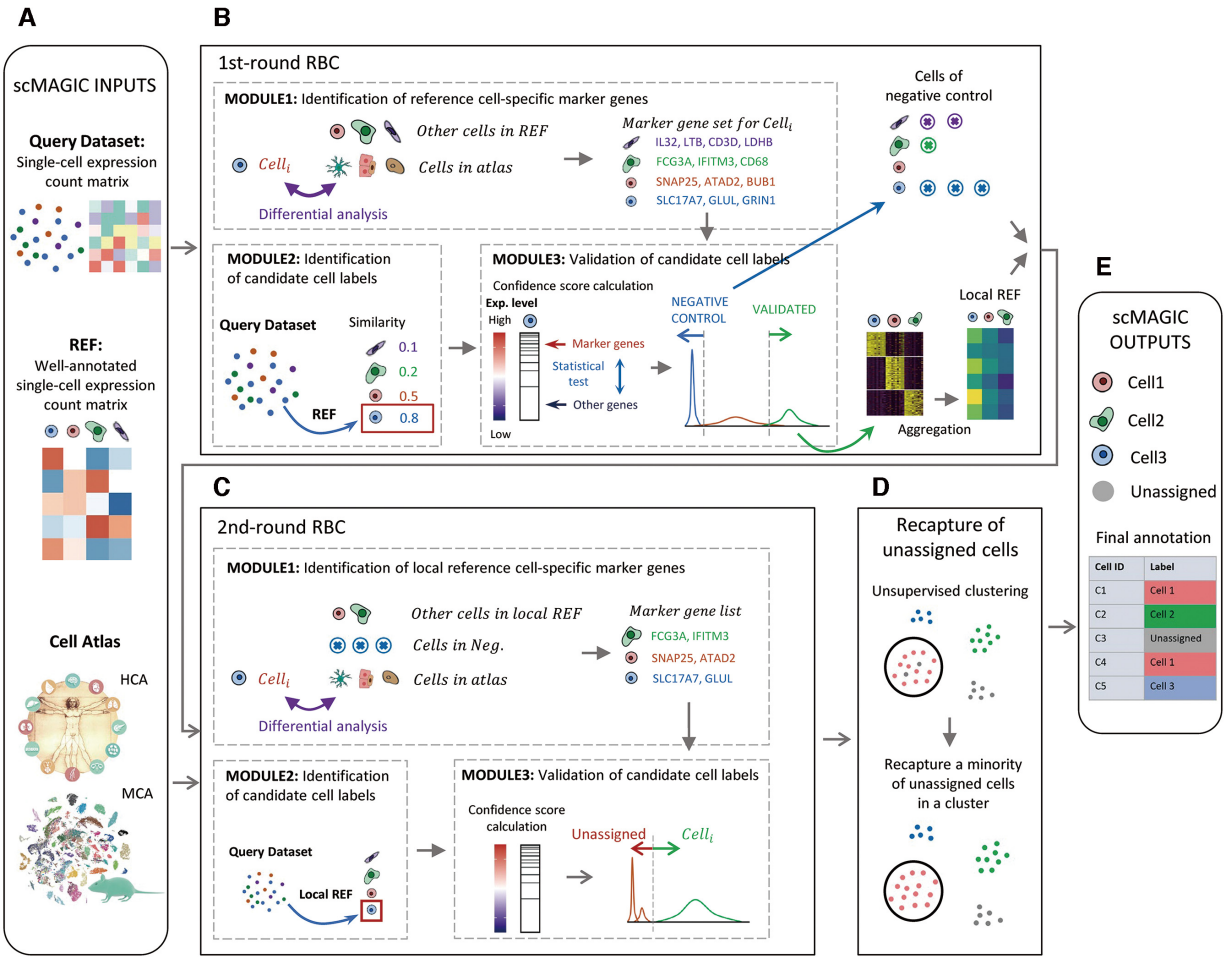
The workflow of scMAGIC. For details about the algorithm design of scMAGIC, refer to the Materials and Methods section.

Users need to provide two expression matrices: a query expression count matrix and a well-annotated reference expression count matrix constructed from scRNA-seq (Figure 1A). In addition to these two matrices, in default scMAGIC also reads the input of an atlas expression profile matrix constructed by aggregating the expression profiles of cells with identical cell types in Mouse Cell Atlas (MCA) or Human Cell Landscape (HCL) (Figure 1A). The row names in these matrices correspond to gene symbols, while the column names correspond to cell barcodes in the query and reference expression count matrices, and correspond to cell type labels in the atlas expression profile matrices. Note that when a reference expression count matrix is provided, users must also provide an annotation file listing the cell type label for each cell.

### The algorithm design of scMAGIC

scMAGIC conducts two rounds of reference-based classification (RBC), followed by a recapturing step, and the workflow of scMAGIC is depicted in Figure 1. There are three main modules in both rounds of RBC: identification of reference cell type-specific marker genes, identification and validation of candidate cell labels for query cells.

#### The three modules in the first-round RBC

We describe in detail these three modules in the first-round RBC (Figure 1B) as the following:

##### Module 1: Identification of reference cell type-specific marker genes

We first convert the reference expression count matrix into an expression profile matrix by aggregating the expression profiles of single cells with the same cell label. For each cell type, we identify the top 20% highly expressed genes and then use *limma* (22) to select from these genes those that are significantly up-regulated with respect to other reference cell types (fdr *P* value > 0.05). Next, we use *RUVSeq* (23) to remove the batch effect between the reference and the atlas expression profile matrix and then use *limma* to further identify from the selected genes those that are significantly up-regulated with respect to the cell types in the atlas expression matrix (fdr *P* value > 0.001). *RUVSeq* has been reported as an effective tool to adjust the technical effects across different bulk RNA-seq expression profiles (24). Here, the reference and the atlas expression profile matrix are both aggregated from cells with the same cell type labels and can be regarded as bulk expression profiles. We evaluate *RUVSeq*’s performance on removing the batch effect by combining one mouse tissue profile and two human tissue profiles with the corresponding MCA and HCL atlas expression profiles, respectively. We find that most reference cell types are merged together with their corresponding cell types in the atlas by using *RUVSeq*, as visualized in the UMAP plots (Supplementary Figure S1), demonstrating its efficacy. Finally, the genes satisfying all above criteria are selected as the marker genes specific for the reference cell type.

##### Module 2: Identification of candidate cell labels

We first identify the top *N* (default is 2000) variable genes in the reference expression profile matrix using the function ‘FindVariableFeatures’ of *Seurat* (25). Then, for each query cell we calculate a similarity score to each reference cell type based on the correlation of the expression profiles of the variable genes, and select the most similar one as its candidate cell label. Here, Kendall’s tau coefficient is selected as the similarity metric because it is based on the relationships between pairs of genes, and is therefore more robust against the batch effects between the query and the reference expression matrix. It is computed by using ‘cor.fk’ in R (‘pcaPP’ package).

##### Module 3: Verification of candidate cell labels

We first group query cells according to their candidate cell labels. For each query cell in a group, we conduct a Wilcoxon rank sum test to inspect whether the expression values of the marker genes corresponding to the cell type of the group are significantly higher than the other genes and convert the *P*-value into a confidence score by negative log transformation. Then, we decompose the density distribution of the confidence scores into several Gaussian distributions using the package *mclust* (26) in R. The labels of all query cells belonging to the sub-distribution with the greatest confidence scores are considered to be validated. In addition, those cells with confidence scores less than a pre-defined threshold (default is 5) are considered as the negative controls for the cell label. The selected negative controls will be used in the second-round RBC to increase the specificity of the identified cell-specific marker genes in discriminating false positive predictions. In the end, we construct a local reference expression count matrix by using only those confidently validated query cells and will use it as the reference in the second-round RBC. Note that the original reference expression matrix will not be used in the second-round RBC. For each cell type label in the local reference expression matrix, we also construct a negative control expression matrix from the query single cells defined as its negative controls. When the atlas expression matrix is used as the reference, we require each local reference cell label have at least 10 validated query cells, to reduce false positive assignments.

#### The differences of the three modules in the second-round RBC

In the second-round of RBC, the original reference expression count matrix is replaced by the local reference expression count matrix produced from the first-round RBC. Below, we describe the differences of the three modules in the second-round of RBC (Figure 1C).

##### The module 1 in the second-round RBC

We use the local reference expression count matrix to identify genes that are up-regulated in a given local reference cell type using the function ‘Findmarkers’ (with fold change > 1.5 and Bonferroni-corrected *P* values < 0.05) in *Seurat*. We then use ‘Findmarkers’ to select from these genes those that are up-regulated with respect to cells in the negative control expression matrix specific for this cell type. Next, we obtain the local reference expression profile matrix by aggregating the expression profiles of cells with the same label and following the same way as in the first-round RBC to identify from the former selected genes those that are up-regulated with respect to the cell types in the atlas expression matrix. These genes are considered as the marker genes specific for the local reference cell type.

##### The module 2 in the second-round RBC

Because there are now no batch effects between the local reference and the query expression matrix, we use multinomial metric as the similarity metric. Multinomial metric describes the probability of observing an expression profile (a query cell) given another expression profile (a local reference cell type) based on multinomial distribution and is calculated using ‘dmultinom’ in R.

##### The module 3 in the second-round RBC

After decomposing the density distribution of confidence scores into several sub-distributions, we inspect the mean of each sub-distribution. If the mean is less than a pre-defined threshold (default is 5), we then label those query cells belonging to this sub-distribution as ‘unassigned’. Otherwise, we consider the query cells of the sub-distribution validated.

#### Recapturing ‘unassigned’ cells by unsupervised clustering

To recapture those query single cells that might be labelled incorrectly as ‘unassigned’, we conduct unsupervised clustering of query cells using the function ‘FindClusters’ in *Seurat* with 50 principal components (PCs) and the resolution set at 3. If majority of cells in a cluster are labeled with the same cell type and if the ‘unassigned’ cells account for <20% of cells in this cluster, we recapture these ‘unassigned’ cells and label them with the cell type of this cluster (Figure 1D).

### Speeding up the computation by scMAGIC

When there are >5000 query cells, we do the followings to speed up the computation. We first cluster query cells using ‘FindClusters’ in *Seurat* with 50 PCs and the resolution of 3. Then, we apply *k*-means algorithm to divide each cluster into *k* sub-clusters, where $( {k = {\rm cell}\ {\rm num}\ {\rm in}\ {\rm the}\ {\rm cluster}/m} )$ and $( {m = {\rm total}\ {\rm cell}\ {\rm num}/3000} )$. Cells in each sub-cluster are then merged into one cell, and the query expression count matrix is constructed from the merged cells. After the application of scMAGIC, the cell labels of merged cells are transferred to their corresponding component cells.

### Data collections

All datasets used in this study are downloaded from public databases and are as follows:

- The mouse brain datasets include the primary visual cortex (PVC) dataset by Tasic *et al.* (4), the primary somatosensory cortex (S1) and the hippocampal CA1 region dataset by Zeisel *et al.* (27), the neocortex (including primary visual cortex (VISp) and the anterior lateral motor area (ALM)) dataset by Tasic *et al.* (28), the hypothalamic arcuate-median eminence complex (HArc-ME) dataset by Campbell *et al.* (29), the dentate gyrus of the hippocampus (DGH) dataset by Hochgerner *et al.* (30) and the ventricular-subventricular zone (V-SVZ) dataset by Mizark *et al.* (31), which can be downloaded from NCBI’s GEO database with the accession number GSE71585, GSE60361, GSE115746, GSE93374, GSE95315 and GSE109447, respectively.
- The mouse pancreas dataset is from the study by Baron *et al.* (5), and its accession number in GEO database is GSE84133.
- The mouse duodenum dataset is from the study by Haber *et al.* (32), and its accession number in GEO database is GSE92332.
- The human pancreas datasets consist of four datasets from the studies of Baron *et al.* (5), Muraro *et al.* (33), Xin *et al.* (34) and Segerstolpe *et al.* (35), and their accession numbers are GSE84133, GSE85241 and GSE81608 in NCBI’s GEO database, and E-MTAB-5061in EBI ArrayExpress database, respectively.
- The human PBMC datasets include three studies by Ding *et al.* (6), Butler *et al.* (25) and Zheng *et al.* (36) and can be downloaded from GEO database with the accession number GSE132044, package *SeuratData* and https://support.10xgenomics.com/single-cell-gene-expression/datasets, respectively.
- The CellBench 10X dataset and the CellBench CEL-Seq2 dataset that are from the study by Tian *et al.* (37) and are both the mixture of five human lung cancer cell lines. In that study, the CellBench 10X dataset is obtained from GSM3618014, and the CellBench CEL-Seq2 dataset is obtained and concatenated from three datasets (GSM3618022, GSM3618023 and GSM3618024).
- The Tabula Muris dataset is from The Tabula Muris Consortium (38), and its accession number in GEO database is GSE109774.
- Both the MCA and the HCL atlas expression data are downloaded from NCBI’s GEO database with the accession number GSE108097 (8) and GSE134355 (7), respectively.

The above datasets are selected to be either the reference or the query datasets. For details about the reference and the query datasets in the 86 benchmark tests corresponding to the three situations and the four exploratory studies, refer to Supplementary Table S1. The cell labels of the above datasets are standardized for the purpose of performance evaluations. For details about the standardization of the cell labels, refer to Supplementary Tables S2–68.

### Performance assessment

We use the following four metrics—accuracy, balanced accuracy, labeled accuracy and labeled balanced accuracy to assess the performance of a method. Accuracy is defined as the proportion of query cells with correctly assigned labels and is calculated as:

$$\begin{equation*}{\rm Accuracy }= \frac{1}{n}\mathop \sum \limits_{i = 1}^n \ \mathbb{I}\left( {{{\hat{y}}_i} = {y_i}} \right)\end{equation*}$$

where $n$ is the number of query cells, ${\hat{y}_i}$ and ${y_i}$ are the predicted and the true cell type of $cel{l_i}$, respectively. Note that the true cell types of query cells whose cell types are not covered by the reference are defined as ‘unassigned’. Balanced accuracy is defined as the accuracy averaged across query cell types and is calculated as:

$$\begin{equation*}{\rm Balanced}\ {\rm Accuracy }= \frac{1}{m}\mathop \sum \limits_{i = 1}^m \ {\rm Accurac}{y_i}\end{equation*}$$

where $m$ is the number of query cell types, $Accurac{y_i}$ is the accuracy calculated for all single cells with the *i*th cell type. Note that ‘unassigned’ is also considered a cell type.

Labeled accuracy and labeled balanced accuracy are calculated without unassigned cells.

In real practice, users usually pay more attention to the identity of cell clusters than individual cells. Here, we apply the following approach to assign cell labels to cell clusters using an RBC method. For a given query dataset in a benchmark test, we conduct unsupervised clustering using the function ‘FindClusters’ in *Seurat* with 50 PCs and the resolution set at 3 to generate cell clusters. After applying an RBC method to annotate all query cells, we simply label each cell cluster with the cell label assigned to most cells by the method in the cluster. To assess the performance of different RBC methods at the cell cluster level, we introduce the cluster accuracy defined as the proportion of clusters with correctly assigned cell labels as follows:

$$\begin{equation*}{\rm Accurac}{y_{{\rm cluster}}} = \frac{1}{N}\mathop \sum \limits_{i = 1}^N \ \mathbb{I}\left( {{{\hat{c}}_i} = {c_i}} \right)\end{equation*}$$

Where $N$ is the number of cell clusters in the query dataset, and ${\hat{c}_i}$ and $c$ are the predicted and the true cell type of ${\rm cluste}{r_i}$, respectively.

### The Classification Difficulty Index (CDI) to quantitatively measure the degree of difficulty for a reference-based classification task

We reason that the degree of difficulty for a classification task depends on two factors: the batch effect between the reference and the query dataset and the proportion of classifiable query cells whose cell types are covered by the reference dataset in the query dataset. More significant batch effect corresponds to a more difficult classification task, and a higher proportion indicates an easier classification task. Because the batch effect can be measured by the F1 of average silhouette width (ASW) score (39), with a higher $F{1_{ASW}}$ indicating less significant batch effect, here we define a classification difficulty index (CDI) by incorporating the ASW score and the proportion of classifiable query cells as the following:

$$\begin{equation*}CDI = \sqrt[2]{{\frac{{{{\left( {1 - F{1_{ASW}}} \right)}^2} + {{\left( {1 - P} \right)}^2}}}{2}}},\end{equation*}$$

where

$F{1_{ASW}} = \frac{{2( {1 - AS{W_{batch\_norm}}} )( {AS{W_{celltype\_norm}}} )}}{{1 - AS{W_{batch\_norm}} + AS{W_{celltype\_norm}}}}$ (39) and *P* refers to the proportion of classifiable query cells.

### Competing methods and parameter tuning

In the 86 benchmark datasets, scMAGIC is compared with 13 competing methods including sciBet (11), SingleR (9), scmap (10), CHETAH (12), scClassify (13), SingleCellNet (15), scPred (14), scID (16), CaSTLe (17) and SVM_rejection_ (18)), Seurat v4 (19), scSemiCluster (20) and CALLR (21), which are downloaded from https://github.com/PaulingLiu/scibet, https://github.com/dviraran/SingleR, https://github.com/hemberg-lab/scmap-shiny, https://github.com/jdekanter/CHETAH, https://github.com/SydneyBioX/scClassify_analysis, https://github.com/powellgenomicslab/scPred/, https://github.com/pcahan1/singleCellNet/, https://github.com/BatadaLab/scID, https://github.com/yuvallb/CaSTLe, https://github.com/tabdelaal/scRNAseq_Benchmark, https://satijalab.org/seurat/, https://github.com/xuebaliang/scSemiCluster, https://github.com/MathSZhang/CALLR, respectively. SciBet, SingleR, scmap are statistical-metric-based methods, and use multinomial model, spearman coefficient and cosine similarity, respectively, as the similarity measure to determine the most relevant reference cell type for a query cell. CHETAH and scClassify are tree-based methods. CHETAH is based on a hierarchical classification tree, while scClassify is a multiscale classification framework based on the ensemble of hierarchical classification trees. SingleCellNet, scPred, scID, CaSTLe, SVM_rejection_ are machine-learning-based methods and are based on random forest, support vector machine (SVM), linear discriminant analysis, eXtreme Gradient Boosting (XGBoost) and SVM (with a linear kernel), respectively. Seurat v4, scSemiCluster, CALLR are semi-supervised learning methods using anchor-based transfer learning, structural regularized domain adaptation, and the combination of unsupervised and supervised learning, respectively.

The default parameter setting of each competing method may not represent their best performance. Thus, we conduct a rigorous search for the optimal combination of parameters for each competing method. To find a fair way to determine the optimal parameter setting for each method, we construct a representative set of benchmark tests by randomly selecting four benchmark tests from each of the first two situations and one benchmark test from the third situation. We then apply each method to these nine benchmark tests by using different combinations of parameters and select the combination with the greatest mean accuracy as the optimal parameter setting to be used in this study. For six methods (scClassify, CHETAH, scPred, scID, singleCellNet, SingleR), their mean accuracy increases by only <0.02 using the optimal parameter settings. Of the remaining seven methods, four (sciBet, CALLR, scSemiCluster, Seurat v4) have slight increase around 0.02–0.05, and three (SVM_rejection_, scmap-cluster, scmap-cell, CaSTLe) have more significant increase, by using their respective optimal parameter settings.

The optimal parameter settings selected for each of the 13 competing methods are the followings. For scClassify, we select the method ‘HOPACH’ to build the hierarchical tree, ‘KNN’ as the base classifier of ensemble model, the number of neighbors for KNN classifier to be 10, and the top number of selected features to be 50 (Supplementary Figure S2). For sciBet, we select the number of genes with the maximum entropy differences to be 600 (Supplementary Figure S3). For scmap-cluster, we set the threshold of the similarity between a query cluster and a reference cell type to be 0.2 (Supplementary Figure S4). For scmap-cell, we set the number of nearest neighbors to be 1 and the threshold of the similarity between a query cell and a reference cell type to be 0.4 (Supplementary Figure S5). For CHETAH, we use the method ‘complete’ for clustering reference profiles and select the number of the genes for producing a classification tree to be four fifths of the mean number of captured genes (Supplementary Figure S6). For scPred, we select 1000 highly variable genes and 50 PCs to train the model (Supplementary Figure S7). For scID, we set the threshold (natural logarithm of fold change) to be 0.4 for extracting markers from the reference clusters (Supplementary Figure S8). For CALLR, we set ‘μ’ (used to balance the effect of logistic regression term and the spectral clustering term) to be 0.9 (Supplementary Figure S9). For singleCellNet, we select 10 genes and 25 gene pairs per category to train the classification model and set the number of trees to be 1000 (Supplementary Figure S10). For scSemiCluster, we set the dimensions of two hidden layers to be 128 and 64, respectively (Supplementary Figure S11). For SingleR, we use the method ‘de’ to extract genes and score each query cell by using the 80th percentile of correlation values calculated between the query cell and each of the reference cells (Supplementary Figure S12). For CaSTLe, we set the number of highly variable genes, the maximum depth of tree, and the minimum sum of child weights to be 2000, 20 and 50, respectively (Supplementary Figure S13). For Seurat V4, we set the number of PC spaces to be 50, and choose 8, 320, and 48 neighbors to find, filter, and score the anchor cells between the query and the reference datasets, respectively (Supplementary Figure S14). For SVM_rejection_, we use 2000 highly variable genes to train the SVM model and set the threshold of possibility values as 0.1 (Supplementary Figure S15).

In scMAGIC, there are two parameters available for tuning: the number of highly variable genes used to calculate the similarity between the profiles of a query cell and a reference cell type and the threshold of confidence score for selecting query cells, which are 2000 and 5, respectively, in default. We change the number of highly variable genes from 200 to 5000 and the threshold of confidence score from 1 to 15 and find that the performance of scMAGIC is stable when the former is around 600–3000 and the latter is around 3–8 (Supplementary Figure S16). Therefore, we choose to use the default setting in this study.

In the exploratory tasks, we compare scMAGIC with scHCL and scMCA which are cell type identifier based on pearson correlation coefficients (7,8). In this study, scHCL and scMCA utilize HCL and MCA to classify query cells respectively and can be downloaded from https://github.com/ggjlab/scHCL and https://github.com/ggjlab/scMCA.

## RESULTS

### The workflow of scMAGIC

scMAGIC conducts two rounds of reference-based classification (RBC), followed by a recapturing step, to annotate query cells with either a reference cell label, or an ‘unassigned’ label indicating that it does not belong to any cell type in the reference. The workflow of scMAGIC is shown in Figure 1. For details, please refer to the Materials and Methods section.

There are three modules in the first-round RBC: identification of reference cell type-specific marker genes, identification and validation of the candidate cell label for query cells. In the first module, the marker genes of a reference cell type are identified by selecting from those highly expressed genes that are up-regulated in the reference cell type with respect to not only the other reference cell types but also the cell types in the atlas. In the second module, the candidate cell label for a query cell is determined by selecting the most similar reference cell type according to expression profile correlation. In the third module, a confidence score is first calculated for each query cell by running a statistical test using the marker genes corresponding to its candidate cell label; then, the candidate cell label of a query cell is validated based on the distribution of the confidence scores of all query cells with the same label, such that only highly confident query cells are validated. In addition, those query cells whose confidence scores are smaller than a pre-defined threshold are selected as the negative controls specific to the cell label. In the end of the first-round RBC, a local reference expression matrix is constructed from those query cells whose cell labels are validated, and a negative control expression matrix is also constructed for each local reference cell label from their negative control query cells (Figure 1B).

In the second-round RBC, the original reference expression matrix is replaced by the local reference expression matrix, thereby eliminating the batch effects between the reference and the query dataset. The three modules in the second-round RBC are similar to those in the first-round except for the followings. In the first module, a negative control expression matrix specific to a local reference cell type is also included when identifying the reference cell type-specific marker genes. In the second module, a different similarity metric is employed. In the third module, relatively less stringent criterion is applied to validate the candidate cell labels. In the end, the unvalidated query cells are labeled with ‘unassigned’ (Figure 1C).

In the recapturing step, query cells are divided into a large number of small clusters. If majority of cells in a cluster are assigned with the same cell label, then those ‘unassigned’ cells within the cluster are recaptured, and are also assigned with the label (Figure 1D). Finally, scMAGIC outputs the annotations for each query cells with either a cell label or an ‘unassigned’ label (Figure 1E).

### Evaluation of scMAGIC’s performance in three situations

When conducting RBC, it is usually assumed that all cell types in the query dataset are covered by the reference dataset. However, in reality the query dataset may also include cell types that are not covered by the reference dataset. In addition, cross-species RBC is often encountered, in which the query and the reference dataset are produced from different organisms. These three situations bring different levels of challenges to the RBC task. Here, to evaluate scMAGIC’s performance, for these three situations we prepare a total of 86 paired reference-query datasets in which 66 are obtained from the benchmark study by Abdelaal *et al.* (40) and Zhao *et al.* (41). We compare scMAGIC with 13 recently published RBC methods, including three statistical-metrics-based (SingleR, Scmap and sciBet), two tree-based (CHETAH and scClassify), five machine-learning-based (scPred, SingleCellNet, scID, CaSTLe and SVM_rejection_) and three semi-supervised-learning-based (Seurat v4, scSemiCluster and CALLR) methods. Using nine benchmark datasets randomly selected from the three situations, we determine the optimal parameter setting for each method and run the method with the setting throughout the evaluations (for details, see Materials and Methods). We use the following metrics to measure the performance of a method: accuracy (the proportion of query cells with correctly assigned labels), balanced accuracy (the accuracy averaged across query cell types), labeled accuracy (the accuracy after removing ‘unassigned’ query cells) and labeled balanced accuracy (the accuracy averaged across cell type labels assigned by an RBC method after removing ‘unassigned’ query cells). Because users generally also pay attention to the identity of cell clusters (42), we also introduce a new metrics called cluster accuracy (the proportion of query clusters with correctly assigned labels) to evaluate the performances of these RBC methods on assigning cell type labels to cell clusters (for details, see Materials and Methods).

#### The first situation: the cell types of the query dataset are covered by the reference dataset

In this situation, we design three scenarios—cross-validation, cross-sample and cross-platform RBC, to mimic the situation in which there are no, little or significant batch effects between the reference and the query datasets, respectively. In the cross-validation scenario, we select 15 datasets to conduct five-fold cross-validation. In the cross-sample scenario, we select 8 pairs of datasets which are produced from the same tissue and platform but from different samples. In the second scenario, we select 33 pairs of datasets that are produced from the same tissue but using different sequencing platforms. For details about the selected datasets and the relationships of cell types between the reference and the query datasets in the third scenario, see Supplementary Table S1 and Supplementary Tables S2–34, respectively.

**Figure 2.**
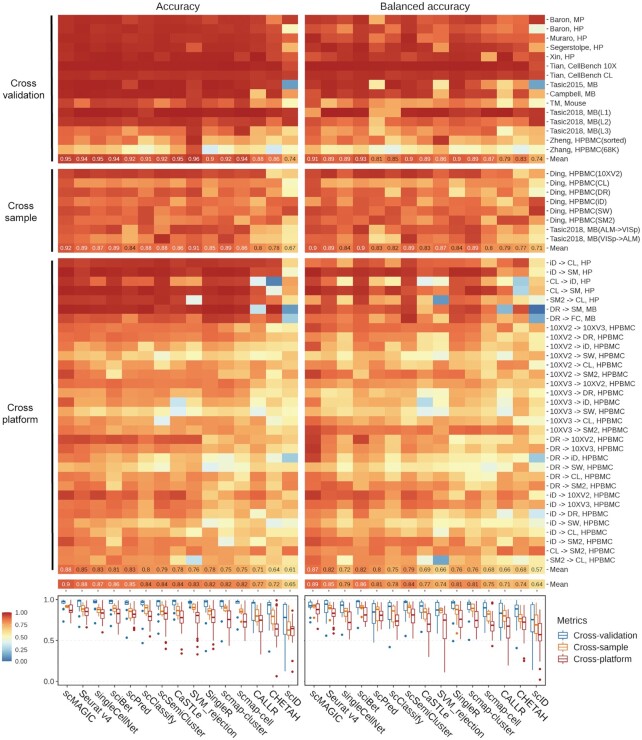
The performance of scMAGIC and 13 competing methods in the first situation. In the cross-validation and cross-sample scenario, we use the first author and the tissue of the study to represent a benchmark test. In the cross-platform scenario, we use the platform of the reference dataset, the platform of the query dataset and the tissue of the two studies to represent a benchmark test. For details about the datasets used in these three scenarios, refer to Supplementary Table S1. The accuracy and the balanced accuracy of the 14 methods are shown in heatmaps. The boxplots of the accuracy and the balanced accuracy of the 14 methods are shown in the bottom respectively. The 14 methods are arranged from left to right according to their mean accuracy in decreasing order; MP, mouse pancreas; HP, human pancreas; MB, mouse brain; HPBMC, human peripheral blood mononuclear cells; iD, inDrops; CL, CEL-Seq2; SM, SMARTer; SM2, Smart-seq2; DR, Drop-seq; FC, Fluidigm C1; 10XV2, 10x Chromium (v2); 10XV3, 10x Chromium (v3); SW, Seq-Well.

Most methods do well in the cross-validation scenario (Figure 2). The averaged accuracy and the averaged balanced accuracy by scMAGIC across the 15 benchmark tests are 0.95 and 0.91, respectively. The averaged accuracy by all competing methods except CALLR, CHETHA and scID is >0.9. Six competing methods (Seurat v4, singleCellNet, sciBet, CaSTLe, scmap-cell and SVM_rejection_) perform comparably (averaged accuracy: 0.94–0.96, averaged balanced accuracy: 0.87–0.93) to scMAGIC (Figure 2). In the cross-sample scenario, scMAGIC achieves an averaged accuracy of 0.92 and an averaged balanced accuracy of 0.9 across the 8 benchmark tests, whereas the averaged accuracy and averaged balanced accuracy by the best competing methods are 0.91 (SVM_rejection_) and 0.9 (sciBet), respectively (Figure 2). In the cross-platform scenario, scMAGIC achieves an averaged accuracy of 0.88 and an averaged balanced accuracy of 0.87 across the 33 benchmark tests, whereas the averaged accuracy and averaged balanced accuracy by the best competing methods are 0.85 (Seurat v4) and 0.82 (Seurat v4 and sciBet), respectively (Figure 2). Overall, scMAGIC ranks the best among all methods, with an averaged accuracy and an averaged balanced accuracy of 0.9 and 0.89, respectively, across the 56 benchmark datasets in the three scenarios of the first situation (Figure 2). We also compare the 14 methods at the cluster level. scMAGIC achieves an averaged cluster accuracy of 0.9, while the averaged cluster accuracy by the best competing method is 0.86 (singleCellNet, sciBet, Seurat v4) (Supplementary Figure S17). Because the first situation is relatively easy, most methods do well, and the advantage of scMAGIC over the competing methods is not very significant despite that it ranks the best among all methods in this situation.

#### The second situation: the query dataset includes cell types not covered by the reference dataset

In this situation, we also design two scenarios to evaluate the impacts of batch effects on the classification performance. In the first scenario, the reference datasets are sequenced with high-depth by technologies such as SMARTer, Smart-seq2 or CEL-Seq2 (>2500 genes captured per cell in average), while the reference datasets in the second scenario are sequenced with low-depth by technologies such as Microwell-Seq or Seq-Well (<650 genes captured per cell in average). These two scenarios are therefore named as the high- and low-depth reference scenario, respectively, and the low-depth reference scenario apparently represents the scenario with more significant batch effects. The high- and low-depth reference scenarios include 15 and 11 benchmark tests, respectively, and in each benchmark test, the query dataset includes some cell types not covered by the reference dataset (for details, see Supplementary Tables S35–60).

**Figure 3.**
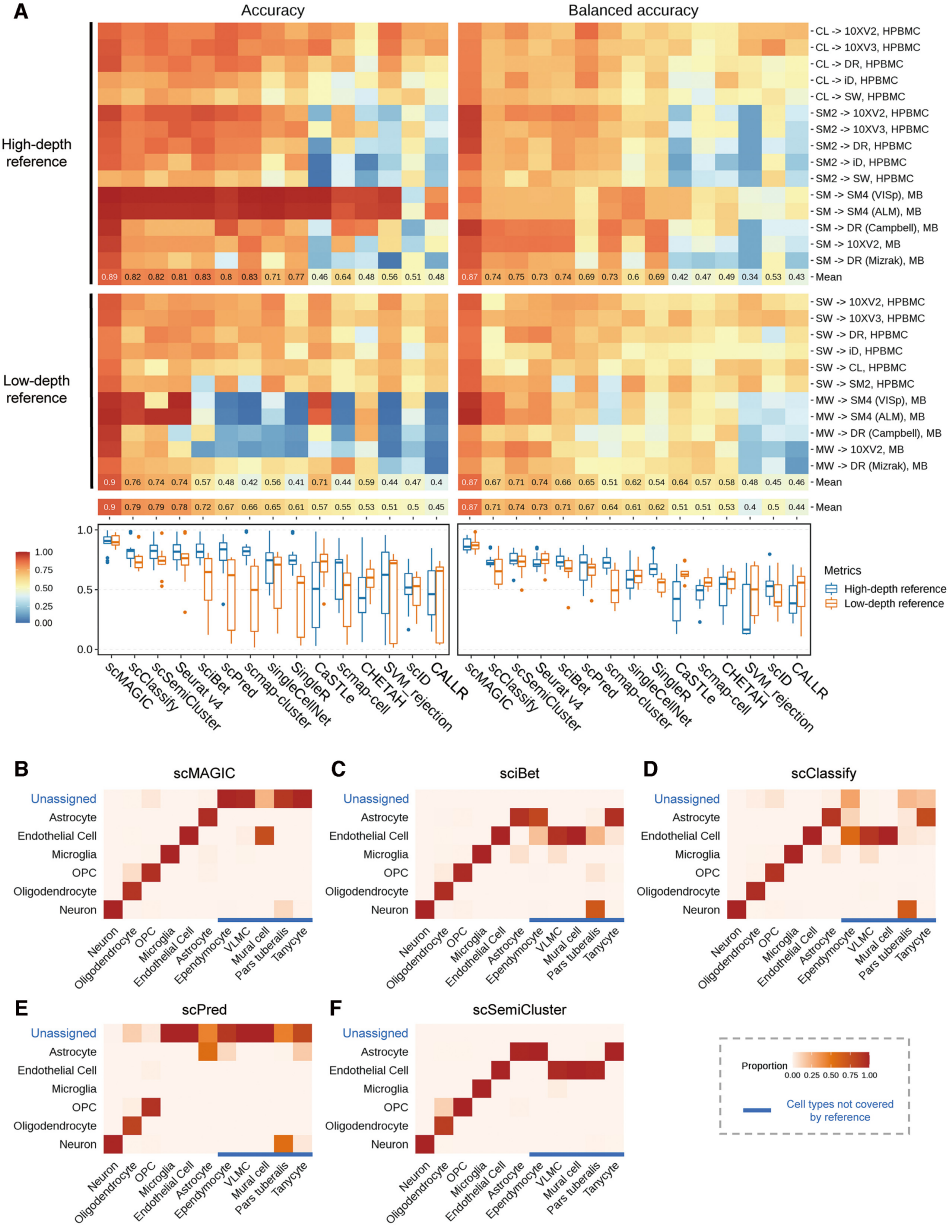
The performance of scMAGIC and 13 competing methods in the second situation. Panel (**A**) is organized in the same way as Figure 2 except that the two scenarios are the high-depth and the low-depth reference scenarios, respectively. Here, a benchmark test is represented by the platforms of the reference and the query dataset and the tissue of the two studies. For details about the datasets used in this situation, refer to Supplementary Table S1. Panels (**B–F**) show the heatmaps of the confusion matrix of scMAGIC, sciBet, scClassify, scPred and scSemiCluster, respectively, in the high-depth reference scenario, with the mouse datasets profiled by SMARTer and Drop-seq (Campbell) as the reference and query dataset respectively. In a confusion matrix, the row and column names correspond to the reference cell labels (including the ‘unassigned’ label) and the query cell labels, respectively, while the element represents the proportion of a specific type of query cells that are assigned with a specific reference cell label; HPBMC, human peripheral blood mononuclear cells; MB, mouse brain; OPC, oligodendrocyte precursor cell; VLMC, vascular and leptomeningeal cell. The abbreviations of the platforms are the same as in Figure 2.

In the high-depth reference scenario, scMAGIC achieves an averaged accuracy and an averaged balanced accuracy of 0.89 and 0.87, respectively, across the 15 benchmark tests (Figure 3A). In the low-depth reference scenario, scMAGIC still achieves an excellent performance, with an averaged accuracy and an averaged balanced accuracy of 0.9 and 0.87, respectively, across the 11 benchmark tests (Figure 3A). In contrast, the competing methods’ performance is not only significantly worse but also significantly affected by the batch effects. In the high-depth reference scenario, the averaged accuracy and the averaged balanced accuracy by the best competing method are 0.83 (sciBet and scmap-cluster) and 0.75 (scSemiCluster), respectively (Figure 3A). In the low-depth reference scenario, these numbers drop to only 0.76 (scClassify) and 0.74 (Seurat), respectively, and the averaged accuracy of sciBet, scPred, scmap-cluster, singleCellNet, SingleR and scmap-cell even drop by >0.2 (Figure 3A). We also inspect the labeled accuracy and the labeled balanced accuracy by removing query cells with ‘unassigned’ labels. The averaged labeled accuracy and the averaged labeled balanced accuracy of scMAGIC are 0.92 and 0.84 in the high-depth reference scenario, respectively, and 0.92 and 0.81 in the low-depth reference scenario, respectively, making it still superior to all competing methods except for singleCellNet that achieves a slightly higher averaged labeled balanced accuracy in the high-depth reference scenario (Supplementary Figure S18A). As for the cluster accuracy, scMAGIC achieves an averaged cluster accuracy of 0.91 in both scenarios, whereas the best competing methods only achieve an averaged cluster accuracy of 0.82 (sciBet and singleCellNet) and 0.78 (scClassify) in the high- and low-depth reference scenario, respectively (Supplementary Figure S19).

Here, we select a benchmark test from the mouse brain in the first scenario as an example to illustrate the performance difference of scMAGIC from the competing methods. We select sciBet, scClassify, scPred and scSemiCluster as the representative method of the statistical-metric-based, the tree-based, the machine-learning-based and the semi-supervised-learning-based RBC methods, respectively, to compare with scMAGIC. The reference dataset (4) includes six cell types, while the query dataset (29) includes 11 cell types in which five are not covered by the reference (for details, see Supplementary Table S47). Those query cells belonging to the six reference cell types are classifiable, while the remaining query cells are unclassifiable. scMAGIC identifies the cell types of the classifiable query cells with very high accuracy (0.99) and very high balanced accuracy (0.97), and also successfully recognizes 97% of unclassifiable query cells (some mural cells are recognized as endothelial cells) (Figure 3B). Both sciBet and scClassify also identify the cell types of the classifiable query cells with very high accuracy (0.99) and very high balanced accuracy (0.96–0.97), but their ability to recognize unclassifiable query cells is poor. sciBet wrongly assigns all unclassifiable query cells with some reference cell labels (Figure 3C), as it assumes that the cell types of the query dataset are all covered by the reference dataset. scClassify identifies only a small portion (19%) of unclassifiable cells (Figure 3D), probably because the hierarchical classification tree built by scClassify is based only on the cell types in the reference dataset, and may not be discriminative enough to recognize unclassifiable query cells. Although scPred recognizes 79% of unclassifiable query cells as ‘unassigned’, it wrongly labels many classifiable query cells (e.g. microglia and endothelial cells) as ‘unassigned’ (Figure 3E), probably because it overfits the reference dataset. scSemiCluster also assumes that the cell types in the query dataset are all covered by the reference dataset. Therefore, although it achieves a high accuracy (0.99) for classifiable query cells, it wrongly assigns all unclassifiable query cells with some reference cell types (Figure 3F).

#### The third situation: cross-species reference-based classification

In this situation, we collect four pairs of mouse and human datasets produced from the same tissues and use the mouse dataset as the reference dataset to annotate the human dataset. Note that for each pair of reference-query datasets, the cell types in the reference and the query datasets may not completely overlap with each other. On the other hand, because a large number of genes are lost during homologous gene conversion, there exist significant batch effects in between the reference and the query datasets. For details about the cell types in the reference and the query datasets, see Supplementary Tables S61–64.

**Figure 4.**
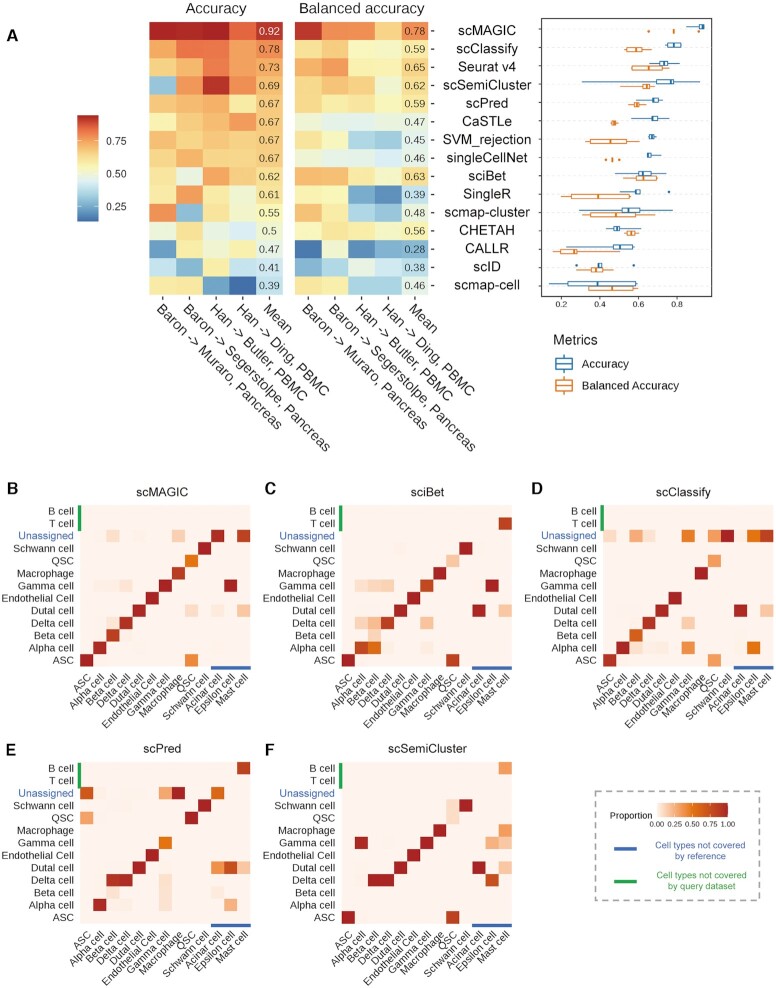
The performance of scMAGIC and 13 competing methods in the third situation. Panel (**A**) shows the heatmaps (left) and the boxplots (right) of the accuracy and the balanced accuracy of the 14 methods. The 14 methods are arranged from top to bottom according to their mean accuracy in decreasing order. In each benchmark test, the reference and the query dataset are produced from the same tissue of mouse and human, respectively. The first authors of the reference and the query dataset and the tissue are used to represent a benchmark test. For details about the datasets used in this situation, refer to Supplementary Table S1. Here, panels (**B–F**) are organized in the same way as (B–F). In this benchmark test, the tissue is pancreas, while the first author of the reference and the query datasets are Baron and Muraro, respectively; ASC, activated stellate cell; QSC, quiescent stellate cell.

In the cross-species RBC, scMAGIC achieves an averaged accuracy and an averaged balanced accuracy of 0.92 and 0.78, respectively, across the four benchmark tests (Figure 4A). In contrast, the averaged accuracy and the averaged balanced accuracy by the best competing methods are only 0.78 (scClassify) and 0.65 (Seurat v4), respectively (Figure 4A). If we consider only query cells assigned with cell labels, the averaged labeled accuracy and the averaged labeled balanced accuracy by scMAGIC are 0.94 and 0.84, respectively, while they are only 0.81 (scClassify) and 0.66 (Seurat v4), respectively, by the best competing method (Supplementary Figure S20A). The averaged cluster accuracy of scMAGIC is 0.9 which is >0.83 (scClassify) by the best competing method (Supplementary Figure S20B).

Here, we select the pair of mouse (5) and human (33) pancreas datasets to illustrate the performance difference of scMAGIC from the competing methods. We also select sciBet, scClassify, scPred and scSemiCluster to compare with scMAGIC. The reference and the query dataset have ten common cell types, while they both include cell types not covered by the other (for details, see Supplementary Table S61). scMAGIC identifies the cell types of almost all classifiable query cells with high accuracy (0.94) except that it wrongly labels some quiescent stellate cells (QSC) as activated stellate cells (ASC). It also correctly recognizes two-thirds of unclassifiable query cells (Figure 4B). In comparison, the performance of the three competing methods is significantly worse. sciBet assigns correct cell labels to only about 40% of classifiable query cells and fails to recognize all unclassifiable query cells (Figure 4C). scClassify assigns 84% of classifiable query cells with a cell label, in which 91% are correct, yet recognizes only fewer than half of unclassifiable query cells (Figure 4D). Although scPred recognizes two thirds of unclassifiable query cells, it wrongly labels 50% of classifiable query cells as ‘unassigned’. However, the accuracy of those classifiable query cells assigned by scPred with cell labels is high (0.92) (Figure 4E). scSemiCluster only correctly classifies about 35% of classifiable query cells and fails to recognize any unclassifiable query cell (Figure 4F).

#### Performance summary of all methods across the three situations

**Figure 5.**
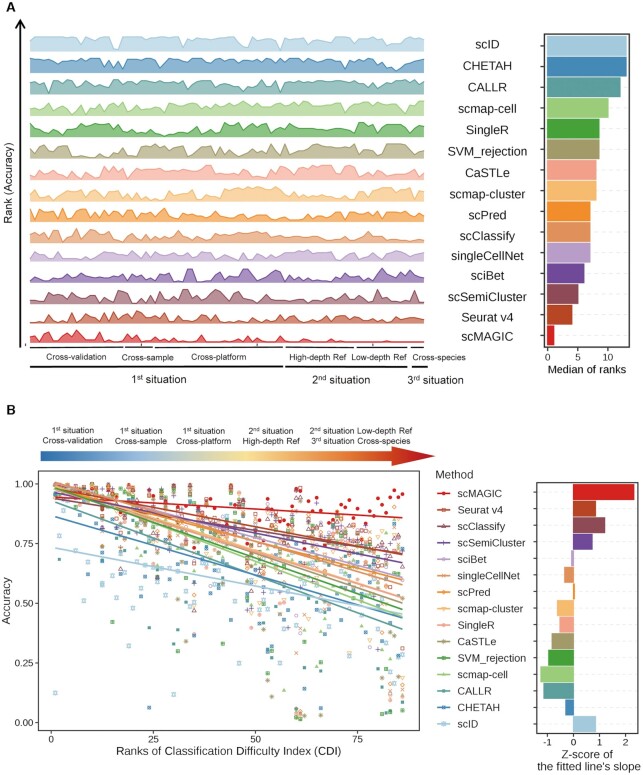
The performance summary of scMAGIC and 13 competing methods across the 86 benchmark tests. Panel (**A**) shows the rank scores and the median of ranks of each of the 14 methods based on their accuracy across the 86 benchmark tests in the left and the right plot, respectively. In the left plot, the height of the ridgelines represents the rank of a method in a benchmark test. The 14 methods are arranged from bottom to top according to their median of ranks in increasing order. (**B**) The left plot shows the accuracy of each of the 14 methods with respect to the ranks of classification difficulty index (CDI) of the 86 benchmark tests. A linear regression model is applied to fit the accuracy to CDI ranks for each method, and the fitted line is plotted. Here, all linear models are statistically significant (*P*-value < 0.001). The 14 methods are arranged from top to bottom according to their mean accuracy in decreasing order. The right plot shows the converted *Z*-score of the slope of the fitted line for each of the 14 methods.

In total, we have conducted 86 benchmark tests representing three situations to evaluate the 14 methods. Here, we summarize the benchmark results by recording the rank of each method in each of the 86 benchmark tests in terms of accuracy and then calculate the median of the ranks across the 86 tests for each method. The advantage of scMAGIC over the competing methods is very significant: scMAGIC achieves a median accuracy rank of 1, while the second-ranked (Seurat v4) and the third-ranked method (scSemiCluster) have a median rank of 4 and 5, respectively (Figure 5A). The benchmark tests in which scMAGIC is not ranked the top mostly belong to the first situation, and scMAGIC actually all performs comparably to the best method in these benchmark tests (Figure 5A). If we use the balanced accuracy to rank all the methods, scMAGIC still achieves a median rank of 1, while the median rank is 4 by the second-ranked methods (sciBet, Seurat v4), respectively (Supplementary Figure S21A). The result based on the cluster accuracy is the same: the median rank by scMAGIC is still 1, in contrast to 4 by the second-ranked methods (sciBet, scSemiCluster and scClassify) (Supplementary Figure S21B).

The 86 benchmark tests belong to three situations that roughly represent different difficulty degree of a classification task. To inspect more directly the performance of different methods in terms of the degree of classification difficulty, here we define a Classification Difficulty Index (CDI) by incorporating the batch effects between the reference and the query dataset measured by the F1 of the average silhouette width (ASW) score (39) and the proportion of classifiable query cells (for details, see Materials and Methods), with a greater CDI score corresponding to a more difficult classification task. We calculate the CDI score for each benchmark test and then rank the 86 tests by their CDI scores. The benchmark tests in the third situation and the low-depth reference scenario of the second situation have the highest CDI ranks, while those in the cross-validation scenario of the first situation have the lowest CDI ranks (Supplementary Figure S22), which are consistent with our expectation on the classification difficulty of these three situations. For each method, we plot its accuracy with respect to the CDI ranks of the 86 tests and use a linear model to fit the accuracy to the CDI ranks (Figure 5B). The averaged accuracy of scMAGIC across the 86 tests is 0.9, in contrast to 0.84 by the best competing method (Seurat v4). In general, all methods’ accuracy decreases with the increase of CDI ranks, i.e., the increase of the degree of classification difficulty. However, scMAGIC’s accuracy decreases only slightly with the increase of CDI ranks, in contrast to much more significant decreases by the competing methods. To further illustrate this tendency, we calculate the slope of each method’s fitted line and convert it into a *Z*-score. The *Z*-score of scMAGIC is 2.32, while the *Z*-scores of the second (Seurat v4), the third (scClassify) and the fourth (scSemiCluster) method ranked by their mean accuracy are 0.85, 1.2, and 0.72, respectively (Figure 5B). This clearly showed that compared to the 13 competing methods, the performance of scMAGIC was much more stable with the increase of CDI ranks. Similar results were obtained by replacing the accuracy with the balanced accuracy or cluster accuracy (Supplementary Figure S23).

### Investigation of the contribution of component algorithm designs on scMAGIC’s performance

There are three special algorithm designs in scMAGIC. The first is the use of a second-round RBC. The second is the addition of an atlas expression matrix when identifying the reference cell type-specific marker genes. The third is the selection of query cells to construct the local expression matrix to be used in the second-round RBC.

**Figure 6.**
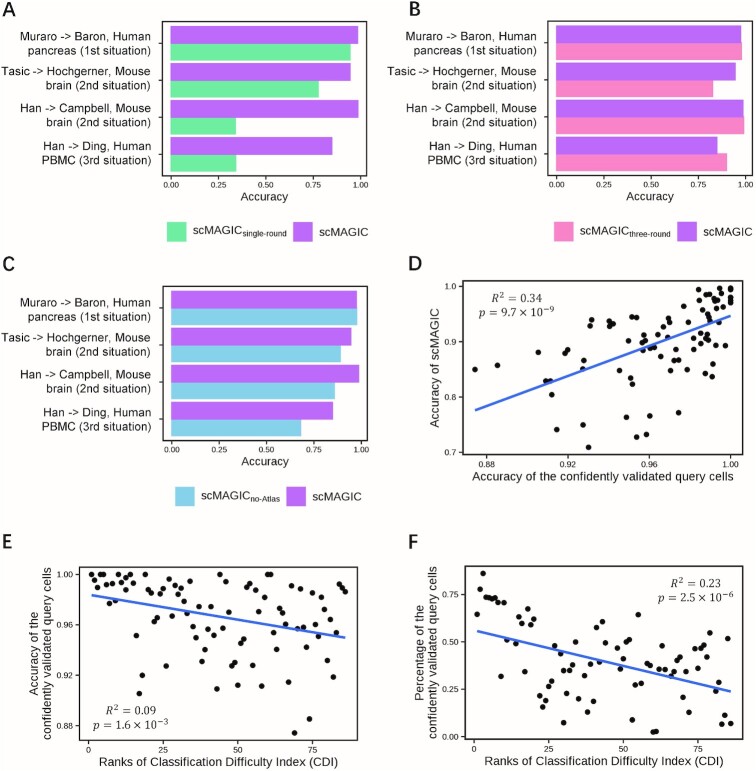
The analysis for scMAGIC’s algorithm designs. Panel (**A**) shows the bar plot of the accuracy of scMAGIC and scMAGIC_single-round_ in four selected benchmark tests. Each benchmark test is represented by the first authors of the reference and the query dataset and the tissue of the query dataset, with the corresponding situation shown in parenthesis. In scMAGIC_single-round_, only one round of RBC is performed. Panel (**B**) is similar to (A) except that scMAGIC is compared with scMAGIC_three-round_ in which three rounds of RBC are performed. Panel (**C**) is similar to (A) except that scMAGIC is compared with scMAGIC_no-Atlas_ in which no atlas expression matrix is included. Panel (**D**) shows the relationship between the accuracy of scMAGIC and the accuracy of the confidently validated query cells in the first-round of RBC. Panel (**E**) shows the relationship between the accuracy of the confidently validated query cells and the increase in CDI ranks. Panel (**F**) shows the relationship between the proportion of the confidently validated query cells and the increase of CDI ranks.

To investigate how the introduction of the second-round RBC contributes to scMAGIC’s performance, we compare scMAGIC with scMAGIC_single-round_ that conducts only one round of RBC in four selected benchmark tests from the three situations. The introduction of the second-round RBC is critical to the performance of scMAGIC. When the batch effects are not significant, e.g. in the two benchmark tests selected from the cross-platform scenario of the first situation and the high-depth reference scenario of the second situation, the accuracy of scMAGIC decreases only slightly by using only one round of RBC. However, the accuracy of scMAGIC drops significantly by using only one round of RBC when there are significant batch effects: in the two benchmark tests selected from the low-depth reference scenario of the second situation and the third situation, the accuracy of scMAGIC is 0.98 and 0.85, respectively, but drops to only 0.33 and 0.34, respectively, by using only one round of RBC (Figure 6A). Nevertheless, adding more rounds of RBC does not further improve scMAGIC’s performance (Figure 6B).

To understand the importance of the inclusion of an atlas expression matrix, we modify scMAGIC to not use the atlas expression matrix (scMAGIC_no-Atlas_). When all query cell types are covered by the reference, e.g. in a dataset selected from the cross-platform scenario of the first situation, the accuracy of scMAGIC_no-Atlas_ is nearly the same as scMAGIC’s (Figure 6C). However, in the other three benchmark tests in which the query datasets include some cell types not covered by the reference dataset, scMAGIC_no-Atlas_ performs evidently worse than scMAGIC does (Figure 6C). Thus, in the presence of unclassifiable query cells, the inclusion of an atlas expression matrix helps to identify marker genes not only more specific to classifiable query cells but also more discriminative to unclassifiable query cells.

The quality of confidently validated cells in the first-round RBC determines the quality of the constructed local reference expression matrix and consequently the classification accuracy in the second-round RBC. In fact, scMAGIC’s classification accuracy is strongly correlated with the accuracy of the confidently validated query cells in the first-round RBC (Figure 6D), further highlighting the necessity of utilizing strict criterion to validate and select the query cells. Because of the implementation of strict selection criterion, the accuracy of confidently validated query cells is above 0.9 in 84 of the 86 benchmark tests, with an mean accuracy of 0.97, and only slightly decreases with the increase in CDI ranks (Figure 6E), thereby ensuring the high accuracy of scMAGIC. However, the cost is that the proportion of confidently validated query cells drops significantly with the increase of CDI ranks: the proportion is around 0.7 in the benchmark tests with the lowest CDI ranks and reduces to around 0.25 in the benchmark tests with the highest CDI ranks (Figure 6F). But the advantage of scRNA-seq is that there are still enough number of query cells left even, making it possible to construct a high-quality local reference expression matrix and consequently producing high-quality predictions. The high quality of the local reference expression matrix constructed in the first-round RBC also explains why the addition of more rounds of RBC would not continue improving the performance of scMAGIC.

### scMAGIC can be used as an exploratory tool for single cell annotation

In an exploratory scRNA-seq study, sometimes it may not be possible for users to find a specific reference dataset. In this case, an atlas expression matrix may be used as the reference to conduct an exploratory annotation of query cells. However, the atlas often includes hundreds of cell types while the query dataset typically includes only several or dozens of cell types. In addition, cells in the atlas are often sequenced at low-depth. These all may bring challenges to the RBC task. To evaluate the usefulness of scMAGIC as an exploratory annotation tool, here we apply scMAGIC to classify two mouse and two human query datasets by using MCA and HCL as the reference datasets, respectively. As MCA and HCL also provide their own annotation tools named scMCA (8) and scHCL (7), respectively, we compare scMAGIC with these two tools.

**Figure 7.**
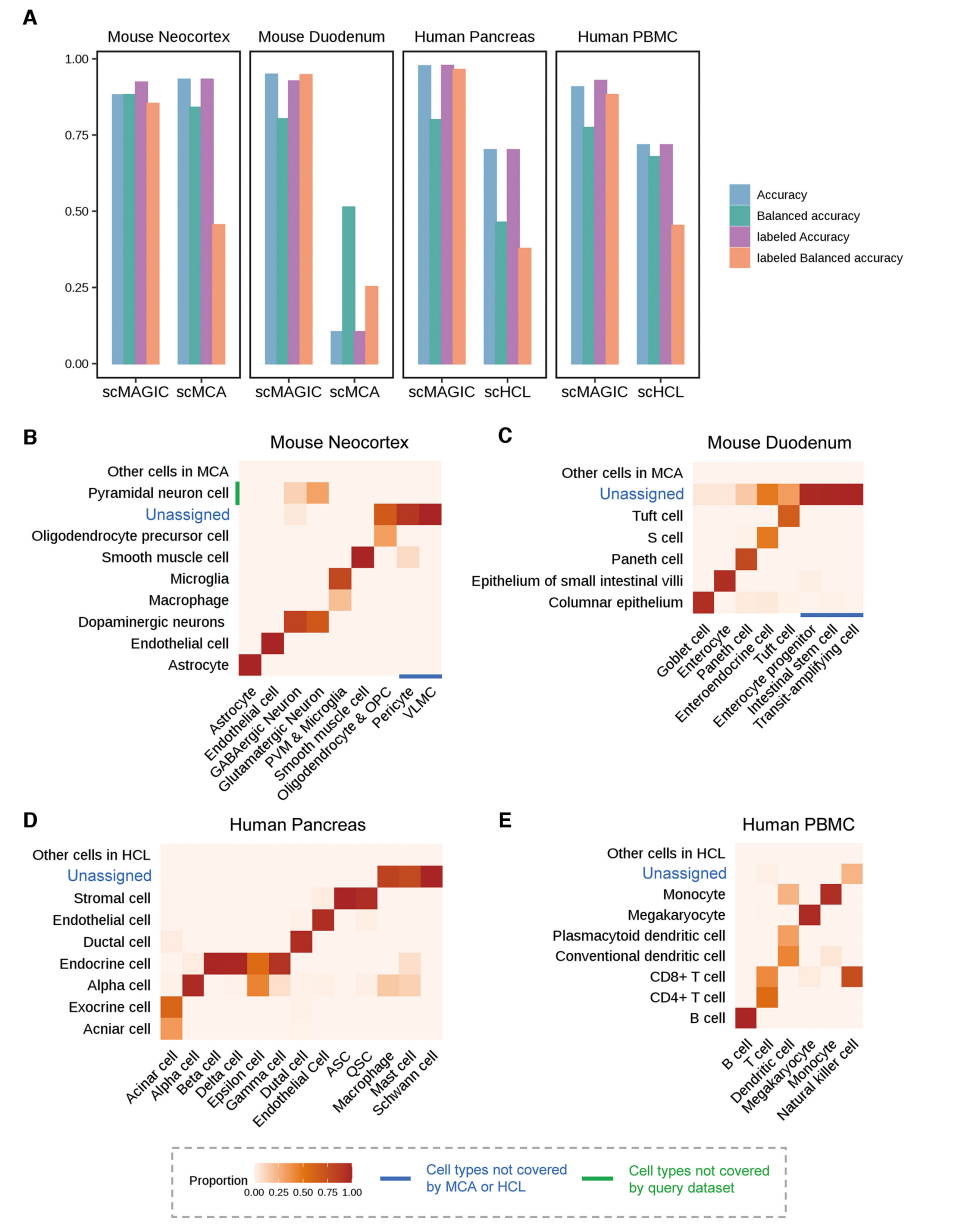
The performance of scMAGIC by using the atlas expression matrix as the reference. Panel (**A**) shows the bar plot of the accuracy, the balanced accuracy, the labeled accuracy and the labeled balanced accuracy of scMAGIC, scMCA and scHCL by using the atlas expression matrix as the reference to annotate four query datasets. For details about the query datasets, refer to Supplementary Table S1. Each benchmark test is represented by the tissue of a query dataset. Panels (**B–E**) show the heatmaps of the confusion matrices of scMAGIC by using MCA (B and C) or HCL (D and E) as the reference to annotate mouse neocortex (B), mouse duodenum (C), human pancreas (D) and human PBMC (E), respectively. Each confusion matrix is arranged in the same way as the confusion matrices in Figure 3B–E. Note that peripheral vascular macrophage (PVM) and microglia are combined into one cell type by the authors, and oligodendrocyte and oligodendrocyte precursor cell (OPC) are also combined into one cell type in (B); VLMC, vascular and leptomeningeal cell; ASC, activated stellate cell; QSC, quiescent stellate cell.

The four query datasets are a mouse neocortex dataset (28), a mouse duodenum dataset (32), a human pancreas dataset (5) and a human PBMCs dataset (6). For details about the matching relationships between the cell labels in the query dataset and in the atlas, refer to Supplementary Tables S65–68. Across these four query datasets, scMAGIC consistently achieves reasonably high accuracy (accuracy: 0.88–0.98, balanced accuracy: 0.77–0.88) (Figure 7A). What’s more, the labeled accuracy and the labelled balanced accuracy by scMAGIC across the four query datasets are 0.92–0.98 and 0.85–0.96, respectively (Figure 7A). Such high labeled accuracy is especially compelling for an exploratory study in the initial round of cell type annotation. In comparison, the performance of scMCA and scHCL is significantly worse. For example, the labeled balanced accuracy of scMCA and scHCL is only 0.25–0.46, and scMAC does very poorly in the mouse duodenum dataset, with an accuracy and an labeled accuracy both around 0.1 (Figure 7A). There are two main reasons why scMCA and scHCL are significantly worse. One is because they both assume all cell types in the query dataset are covered by the atlas, which may not be true, e.g. in the two mouse datasets. Another is because they tend to assign more cell types than needed, leading to poor labeled balanced accuracy. For details about the performance of scMAC and scHCL in these four query datasets, refer to Supplementary Figure S24. Below, we provide more detailed illustrations about scMAGIC’s performance in these four query datasets.

The mouse neocortex dataset (28) consists of nine cell types (for details, see Supplementary Table S65). Among these cell types, pericyte and vascular and leptomeningeal cell (VLMC) are not covered by the MCA, and are assigned by scMAGIC as ‘unassigned’. Astrocytes, endothelial cells, smooth muscle cells (SMC), peripheral vascular macrophages (PVM) and microglia are correctly assigned by scMAGIC with their corresponding labels. Most query cells of GABAergic neuron and glutamatergic neuron are assigned by scMAGIC with the label of ‘dopaminergic neuron’ (these are considered approximately matches because these two types of neurons and dopaminergic neuron all produce neurotransmitters (43)), while a small portion are assigned with the false label of ‘pyramidal neuron cell’. Most query cells of oligodendrocyte and OPC are not recognized by scMAGIC, while a portion of them are correctly assigned with the label of ‘OPC’ (Figure 7B).

The mouse duodenum dataset (32) consists of eight cells types (for details, see Supplementary Table S66). Three of the eight cell types are not included in MCA, and are all assigned by scMAGIC with the ‘unassigned’ labels. Five query cell types are assigned by scMAGIC with cell labels in MCA, in which two (Tuft and Paneth cell) are exact matches. We investigate the relationships between the other three pairs of cell types, and find evidence that they can be considered approximately matches (goblet cell and columnar epithelium (44), enterocyte and epithelium of small intestinal villi (45), and enteroendocrine cell and S cell (46)). However, about 15% of classifiable query cells are recognized as ‘unassigned’ by scMAGIC (Figure 7C).

The cell types in the human pancreas (5) dataset are all classifiable, meaning they are either already included in HCL, or can be approximately matched to some cell labels in HCL (for details, see Supplementary Table S67). In pancreas, acinar cell belongs to exocrine cell (5), alpha cell, beta cell, gamma cell, delta cell and epsilon cell belong to endocrine cell (5), and ASC and QSC belong to stromal cell (47). Therefore, it's correct that acinar cells in the query dataset are labeled by scMAGIC with either acinar cell or exocrine cell, and alpha cells are labeled with either alpha cell or endocrine cell. However, scMAGIC labels three classifiable query cell types (macrophage, mast cell and schwann cell) as ‘unassigned’ (Figure 7D).

The human PBMCs dataset (6) includes six cell types that are all covered by HCL (for details, see Supplementary Table S68). In this dataset, the balanced accuracy by scMAGIC is relatively low. This is because about 25% of Dendritic cell are wrongly labeled as Monocyte, while all NK cells are wrongly labeled (Figure 7E). However, all these other query cells are correctly labeled.

### Running time evaluation

Here, we select the dataset of Hochgerner *et al.* (30) as the reference dataset and the dataset of Campbell *et al.* (28) as the query dataset, and randomly select a portion of query cells to do the classification. Then, we compare the runtimes of the RBC methods investigated in this study, and inspect how they scale when the number of query cells increases. scMAGIC finishes the classification of 20 000 query cells within 6 min, and its runtime does not increases much with the increase of cell numbers when the cell number is over 5000 because of its speed-up setting (for details, see Materials and Methods). There are over half of methods which cannot finish the classification of 20 000 query cells within 6 min, such as scmap-cell, CHETAH, scPred, scID, singleR, scSemiCluster, CaSTLe and CALLR. The runtimes of scmap-cell, singleR, scSemiCluster, CaSTLe and CALLR significantly increase with the increase of cell numbers (Supplementary Figure S25A). We also compare the runtime of scMAGIC with that of scMCA and scHCL in the four query dataset investigated in the exploratory study. scMAGIC’s runtime is around 8–12 min, in contrast to 40–130 min by scMAC and scHCL (Supplementary Figure S25B). Thus, scMAGIC is reasonably efficient, and is well suited for large-scale single cell data analysis.

## DISCUSSION

An underlying assumption of current RBC methods is that the cell types in the query dataset are covered by the reference dataset. When this assumption holds true, i.e. in the first situation in this study, most RBC methods indeed do well. However, in the second and the third situations when the query datasets include unclassifiable query cells whose cell types are not covered by the reference, the performance of most RBC methods is significantly affected. For example, sciBet, a statistical-metrics-based, and scSemiCluster, a semi-supervised-learning-based method, wrongly assign all unclassifiable query cells with some reference cell labels. Another major factor affecting the performance of current RBC methods is the batch effects between the reference and the query datasets, which especially affect those RBC methods that compare query expression profiles directly with reference expression profiles, such as the statistics-based, the tree-based and the machine-learning based RBC methods. Although the semi-supervised-learning-based methods tackle this problem by integrating reference and query cells using different techniques, the significant batch effects may still affect the quality of the integration and consequently its performance. By taking these two factors into consideration, we develop an CDI metric to represent the degree of classification difficulty and find that the performance of all the 13 RBC methods investigated in this study drops evidently with the increase of CDI ranks of the 86 benchmark tests.

scMAGIC, a novel two-rounds RBC method developed in this study, well addresses the above-mentioned challenges. In order to more effectively recognize unclassifiable query cells, scMAGIC adds an atlas expression matrix when identifying the reference cell type-specific marker genes, making the selected marker genes more specific to the reference cell types and consequently more selective to distinguish unclassifiable query cells. As for dealing with the batch effects, scMAGIC innovatively introduces a second-round RBC by replacing the original reference expression matrix with a local reference expression matrix constructed from query cells with verified cell labels and redoing the classification. As the local reference expression matrix belongs to the query expression matrix, there are now no batch effects in the second-round RBC. To some extent, scMAGIC can also be considered as a semi-supervised method because it uses the confidently validated query data to construct the local reference expression matrix to be used in the second-round of RBC. However, what makes it different from other semi-supervised-learning-based methods, such as Seurat v4 and scSemiCluster, is that in scMAGIC, the original reference expression matrix is not used anymore when predicting the cell labels of query cells in the second-round of RBC. In contrast, Seurat v4 and scSemiCluster use not only the query cells but also the reference data as the training set by integrating the reference and the query data into the same space. Although the integration process reduces the batch effects between the query and the reference data, the quality of the integration may still be affected by the batch effects, which will then affect the quality of the final annotations. In scMAGIC, the batch effects can only directly affect the quality of the confidently validated query cells. However, our strict selection criterion has ensured that the confidently validated query cells are of very high quality even for those tests with very high CDI ranks (the mean accuracy of the confidently validated query cells is 0.97 across the 86 tests), and consequently ensures the accuracy of the second-round RBC. Owing to these algorithm designs, scMAGIC consistently achieves excellent performance in the three situations evaluated in this study. It not only correctly assigns reference cell labels to classifiable query cells but also successfully recognizes unclassifiable query cells, and its performance is almost not affected by the batch effects between the reference and the query datasets.

A number of large-scale scRNA-seq studies have constructed the atlas of single cells consisting of hundreds or even thousands of cell types. Theoretically, the atlas can be used as the reference when no specific reference dataset is available, making an RBC method a general-purpose tool for single cell annotation. However, on the one hand, the atlas is still incomplete and a query dataset may include some cell types not covered by the atlas, such as the mouse duodenum dataset (32). On the other hand, cells in the atlas are usually sequenced at low-depth, leading to significant batch effects between the atlas and the query datasets. These are the main reasons why scMCA and scHCL, the tools provided by MCA and HCL, do not work well in the four query datasets investigated in this study. In contrast, scMAGIC achieves reasonably high accuracy by using the atlas as the reference because of its algorithm designs. As such, scMAGIC is of great use not only as a highly accurate RBC method by using a specific reference dataset but also as a general-purpose tool for classifying query cells by using an atlas expression matrix as the reference.

## DATA AVALIBILITY

All single cell gene expression datasets in this study were obtained from their public accessions. The detailed information including the accession codes and publication citations for all datasets can be seen in Materials and Methods.

## CODE AVALIBILITY

scMAGIC can be used in R and its input and output are compatible with Seurat object. The source code is maintained at https://github.com/TianLab-Bioinfo/scMAGIC.

## Supplementary Material

gkab1275_Supplemental_Files
