# Supplementary material for: scMAGIC: accurately annotating single cells using two rounds of reference-based classification
Source: Nucleic Acids Res. 2022 Jan 5;50(8):e43. doi: 10.1093/nar/gkab1275 (PMC9071478; doi:10.1093/nar/gkab1275)
Supplement: gkab1275_Supplemental_Files [file gkab1275_supplemental_files.zip › Supplementary figures.pdf]

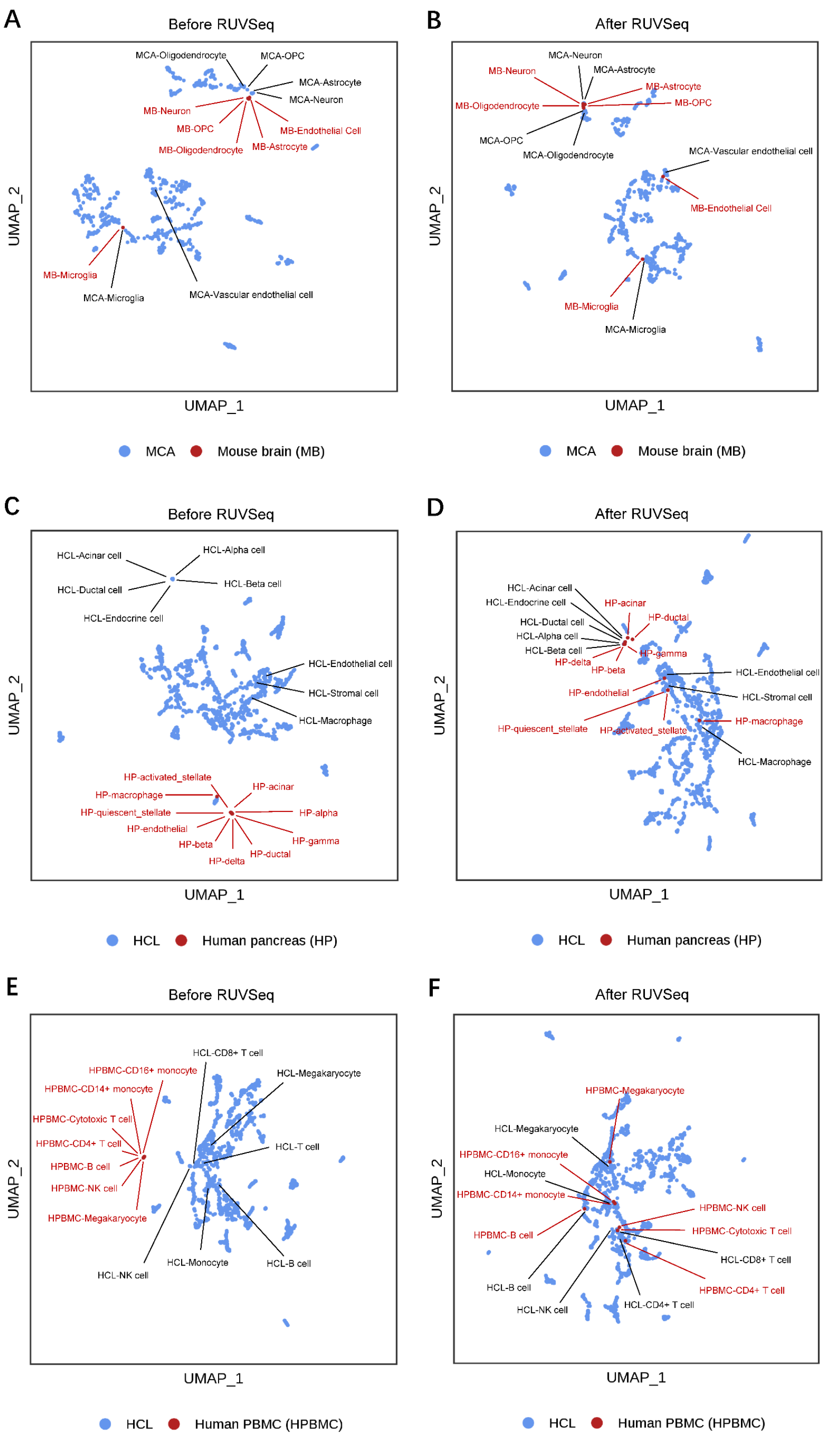

Supplementary Figure S1 The performance of RUVSeq on removing the batch effects between the reference and the atlas expression profiles. (A-B) show the UMAP plots of combining the mouse brain expression profiles from Tasic et al. with MCA expression profiles before **(A)** and after **(B)** the use of RUVSeq, respectively. **(C-D)** show the UMAP plots of combining the human pancreas expression profiles from Segerstolpe et al. with human HCL expression profiles before **(C)** and after **(D)** the use of RUVSeq, respectively. **(E-F)** show the UMAP plots of combining the human PBMC expression profiles from Ding et al. with human HCL expression profiles before **(E)** and after **(F)** the use of RUVSeq, respectively.

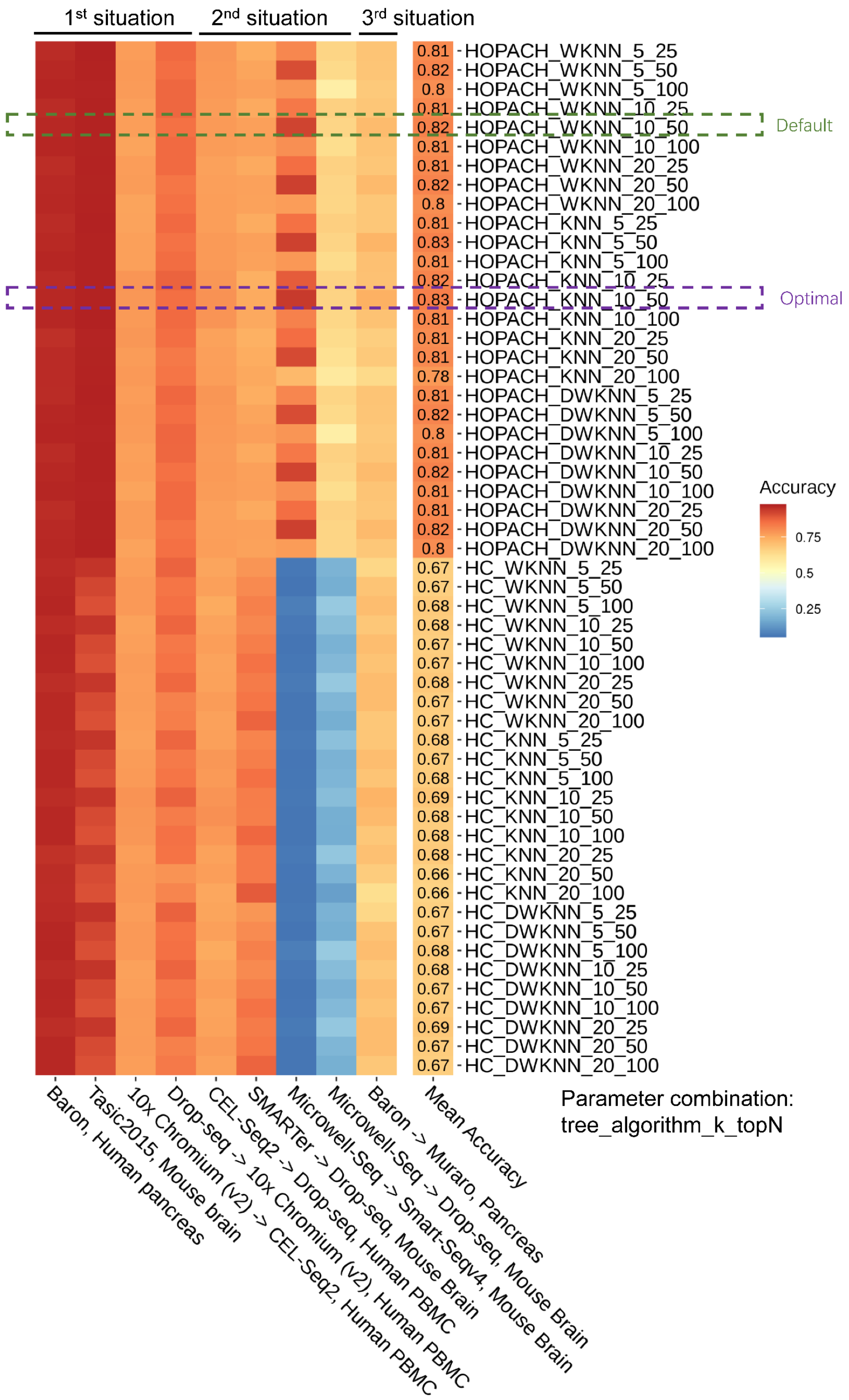

Supplementary Figure S2 The performance of scClassify in nine selected benchmark tests by using different combinations of parameters. There are four parameters for tuning: "tree" corresponds to the method to build the hierarchical tree, "algorithm" refers to the method of base classifier, "k" corresponds to the number of neighbors, and "topN" refers to the top number of features to be selected. The default and the optimal parameter settings corresponding to the greatest mean accuracy are highlighted by green and purple rectangle, respectively.

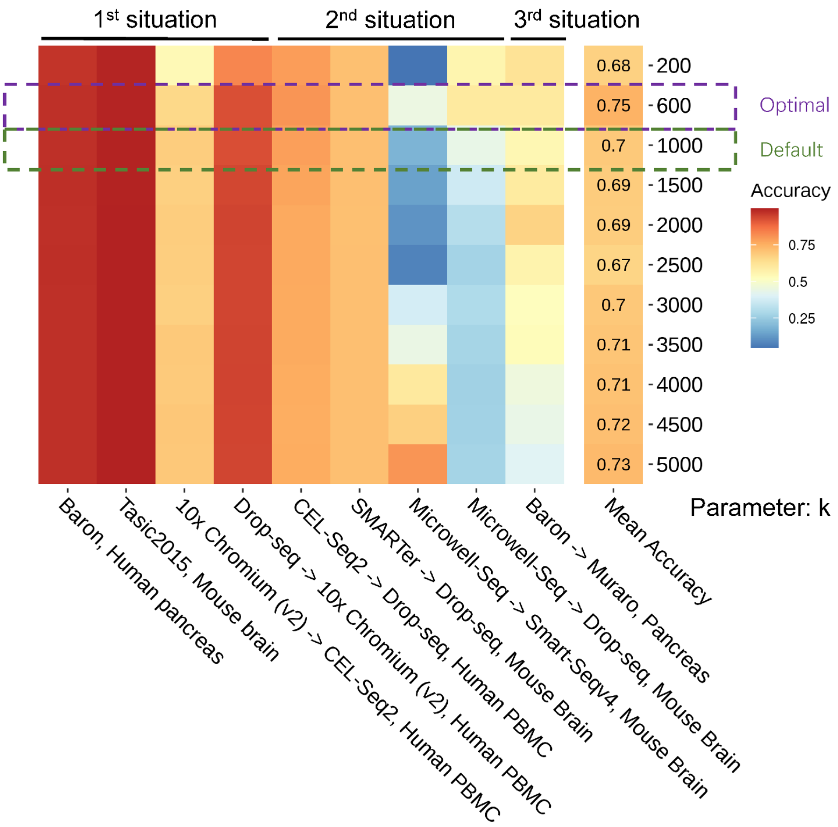

Supplementary Figure S3 The performance of sciBet in nine selected benchmark tests by using different parameters. There is only one parameter for tuning: "k" corresponds to the number of genes with the maximum entropy differences. The default and the optimal parameter are highlighted by green and purple rectangle, respectively.

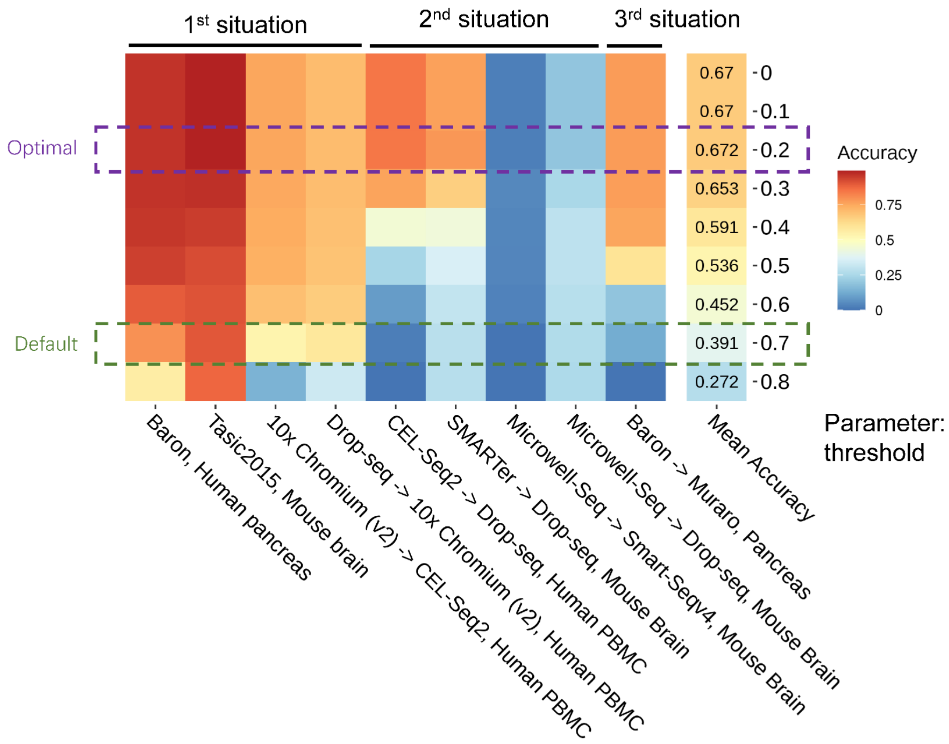

Supplementary Figure S4 The performance of scmap-cluster in nine selected benchmark tests by using different parameters. There is only one parameter in scmap-cluster to tune: "threshold" refers to the threshold of the similarity between a query cluster and a reference cell type. The default and the optimal parameter settings are highlighted by green and purple rectangle, respectively.

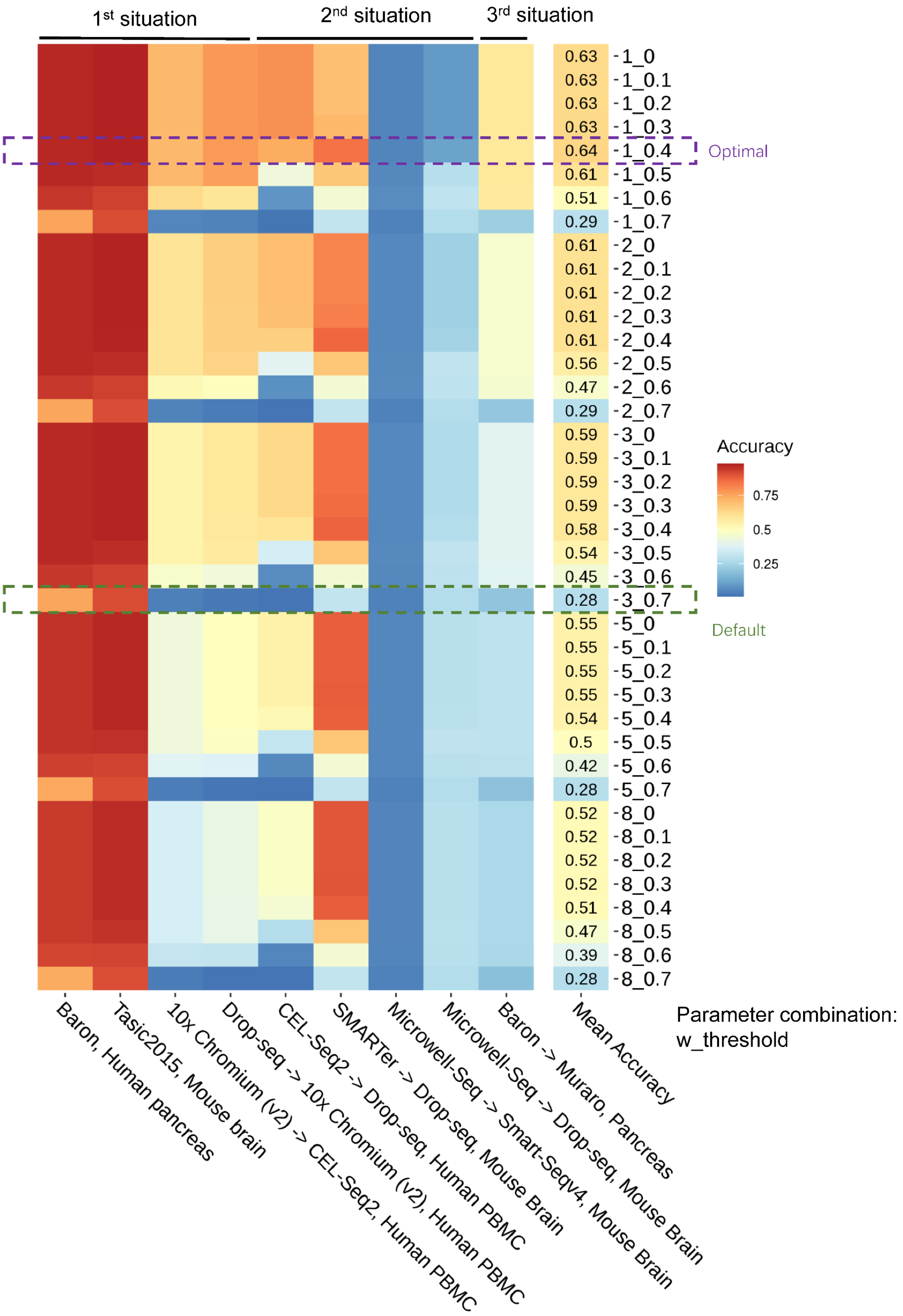

Supplementary Figure S5 The performance of scmap-cell in nine selected benchmark tests by using different combinations of parameters. There are two parameters to tune: "w" refers to the number of nearest neighbors, and "threshold" refers to the threshold of the similarity between a query cell and a reference cell type. The default and the optimal parameter settings are highlighted by green and purple rectangle, respectively.

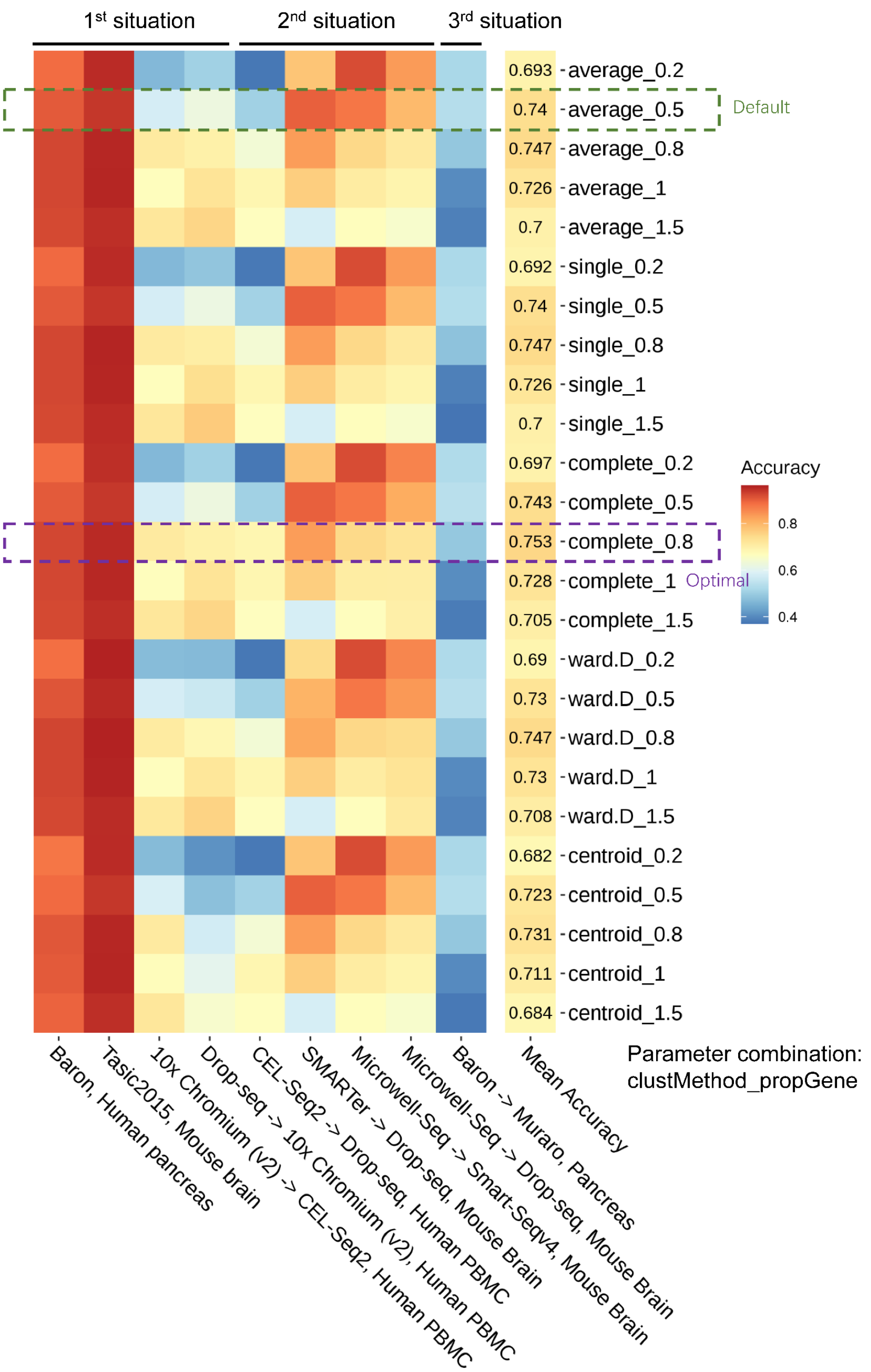

Supplementary Figure S6 The performance of CHETAH in nine selected benchmark tests by using different combinations of parameters. There are two parameters to tune: the first parameter "clustMethod" refers to method used for clustering the reference profiles, and the second parameter "propGene" refers to the ratio of the genes used to produce a classification tree to the mean number of the captured genes. Default and optimal parameter combination are highlighted by green and purple rectangle, respectively.

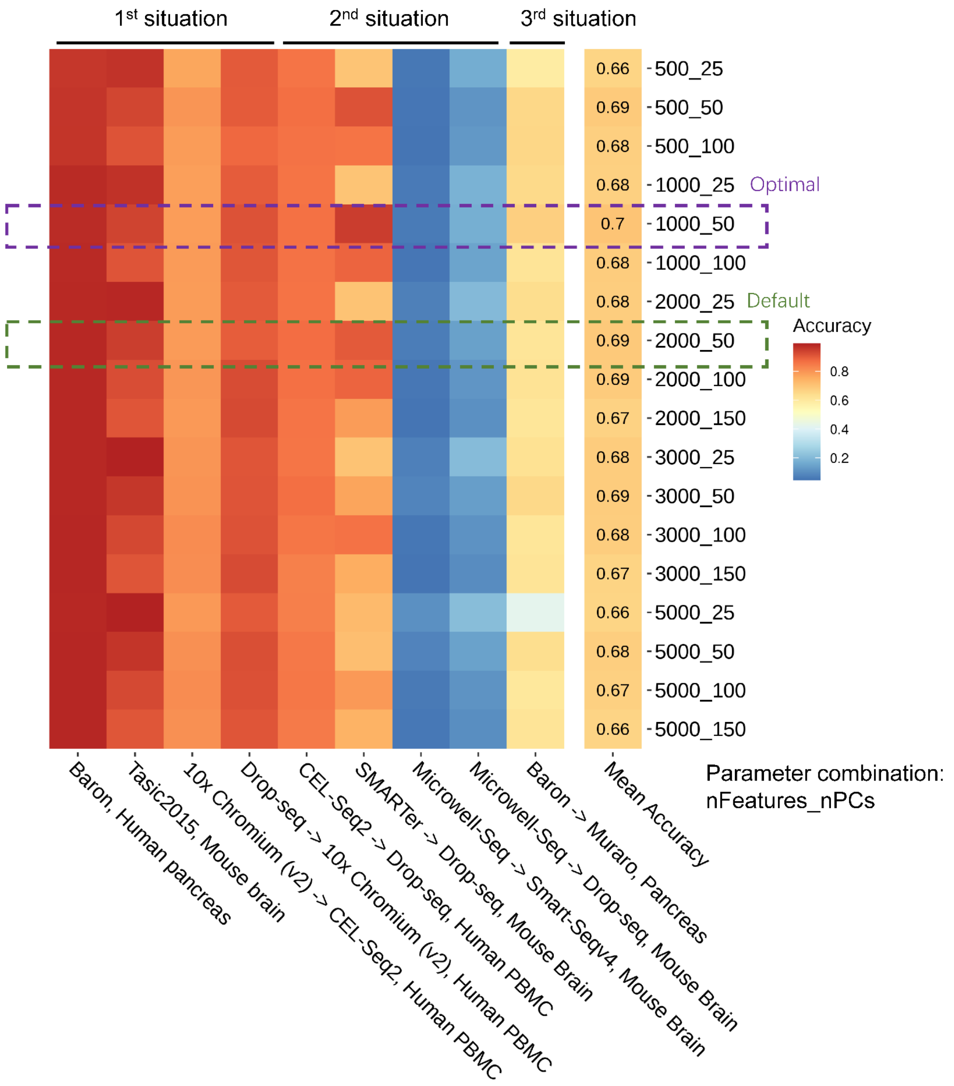

Supplementary Figure S7 The performance of scPred in nine selected benchmark tests by using different combinations of parameters. There are two parameters to tune: "nFeature" refers to the number of highly variable genes, and "nPCs" refers to the number of principle components. Default and optimal parameter combination are highlighted by green and purple rectangle, respectively.

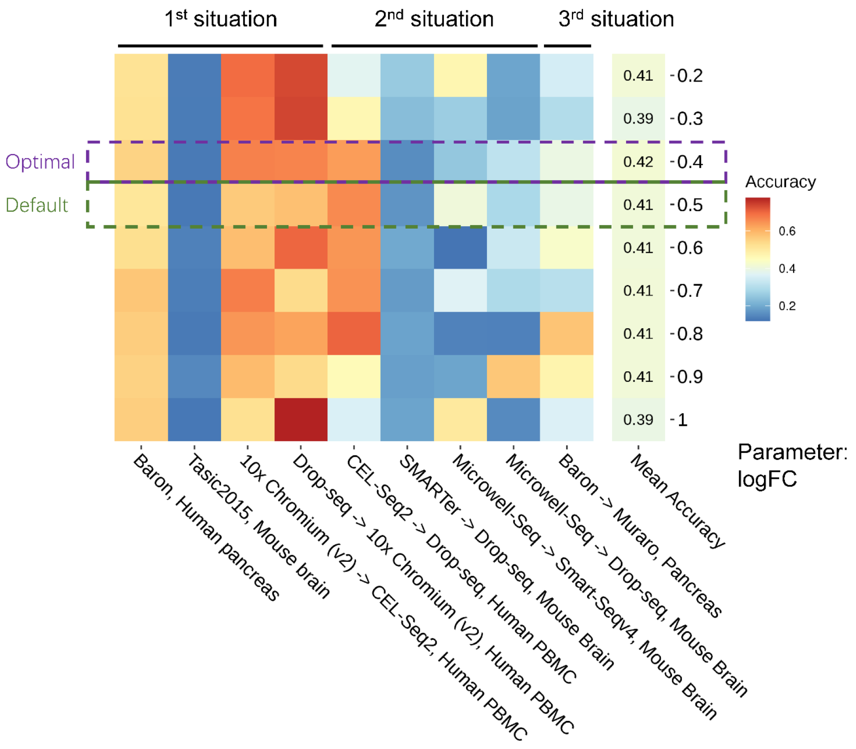

Supplementary Figure S8 The performance of scID in nine selected benchmark tests by using different parameters. There is only one parameter in scID to tune: "logFC" corresponds to the logFC (natural logarithm of fold change) threshold for extracting markers from reference clusters. Default and optimal parameter are highlighted by green and purple rectangle, respectively.

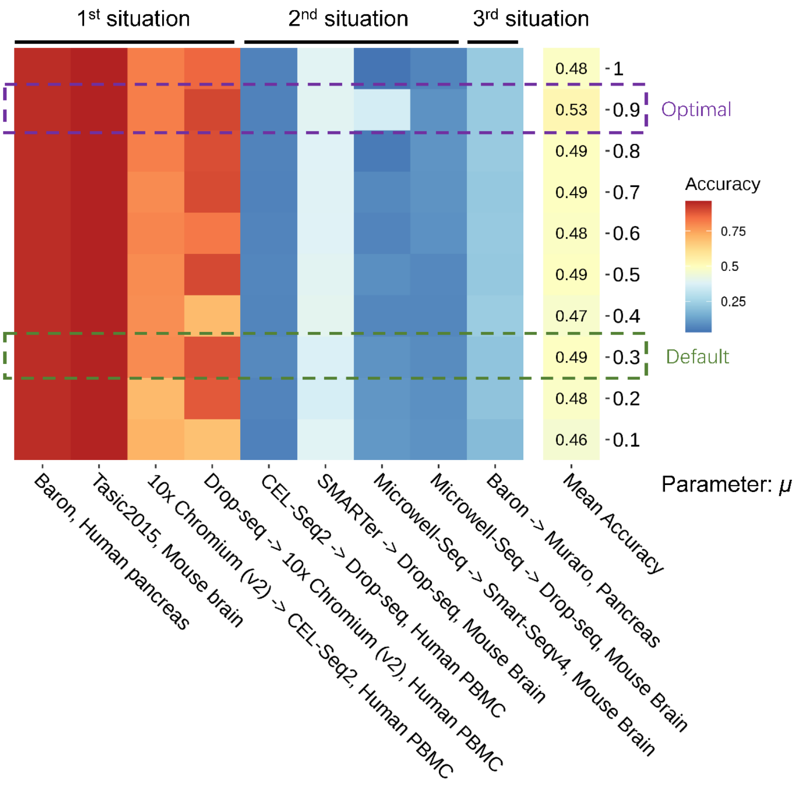

Supplementary Figure S9 The performance of CALLR in nine selected benchmark tests by using different parameters. There is only one parameter to tune: "μ" which is used to balance the effect of logistic regression term and the spectral clustering term. Default and optimal parameter are highlighted by green and purple rectangle, respectively.

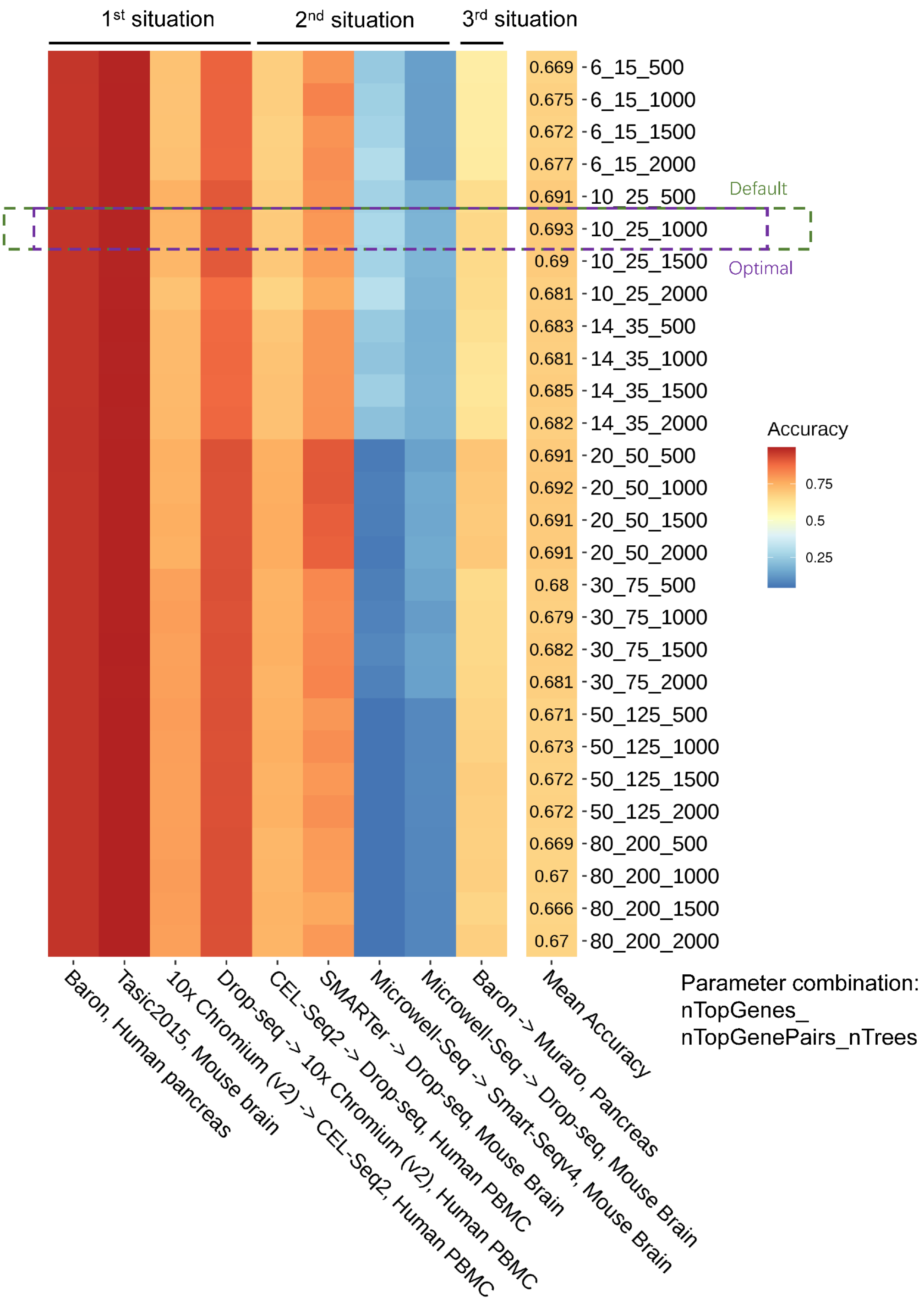

Supplementary Figure S10 The performance of singleCellNet in nine selected benchmark tests by using different combinations of parameters. There are three parameters to tune: “nTopGenes” refers to the number of classification genes per category, “nTopGenePairs” refers to the number of top gene pairs per category, and “nTrees” refers to the number of trees for random forest classifier. Default and optimal parameter combination are highlighted by green and purple rectangle, respectively.

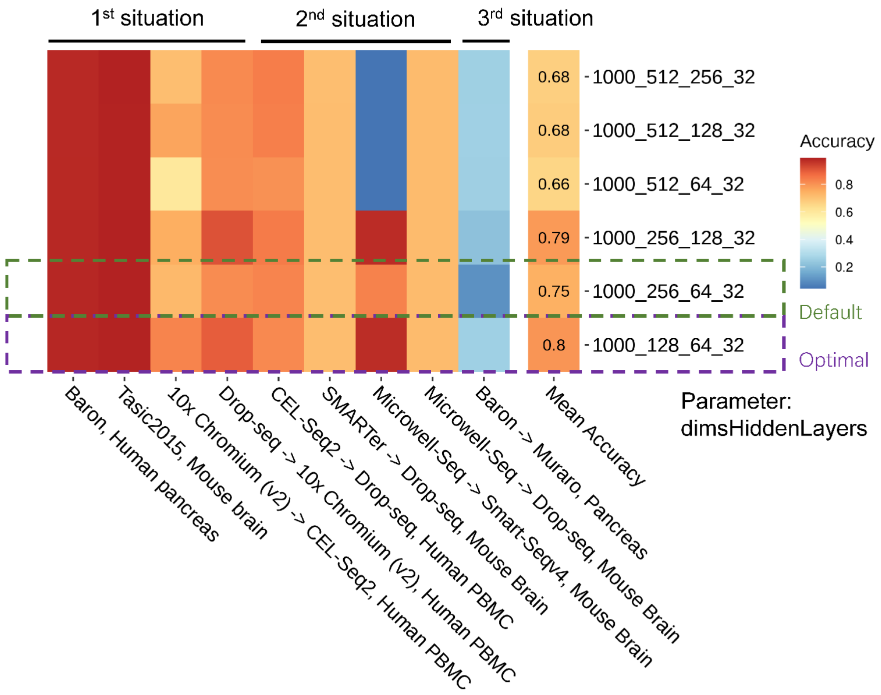

Supplementary Figure S11 The performance of scSemiCluster in nine selected benchmark tests by using different parameters. The dimensions of two hidden layers are tuned for the best performance. Default and optimal parameter are highlighted by green and purple rectangle, respectively.

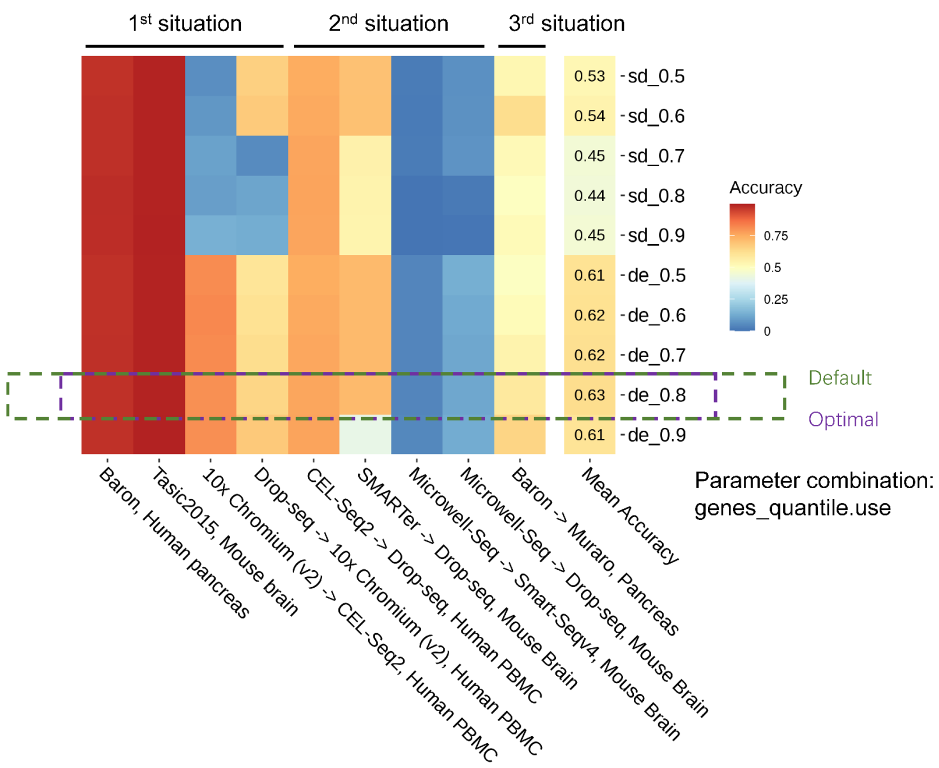

Supplementary Figure S12 The performance of SingleR in nine selected benchmark tests by using different combinations of parameters. There are two parameters to tune: "genes" refers to the method for extracting the genes from the data, and "quantile.use" refers to quantile of correlation coefficients (the similarity between each single query cell and each reference cell type is scored by using 80th percentile of correlation values between the query cell and all reference cells belonging to the reference cell type). Default and optimal parameter combination are highlighted by green and purple rectangle, respectively.

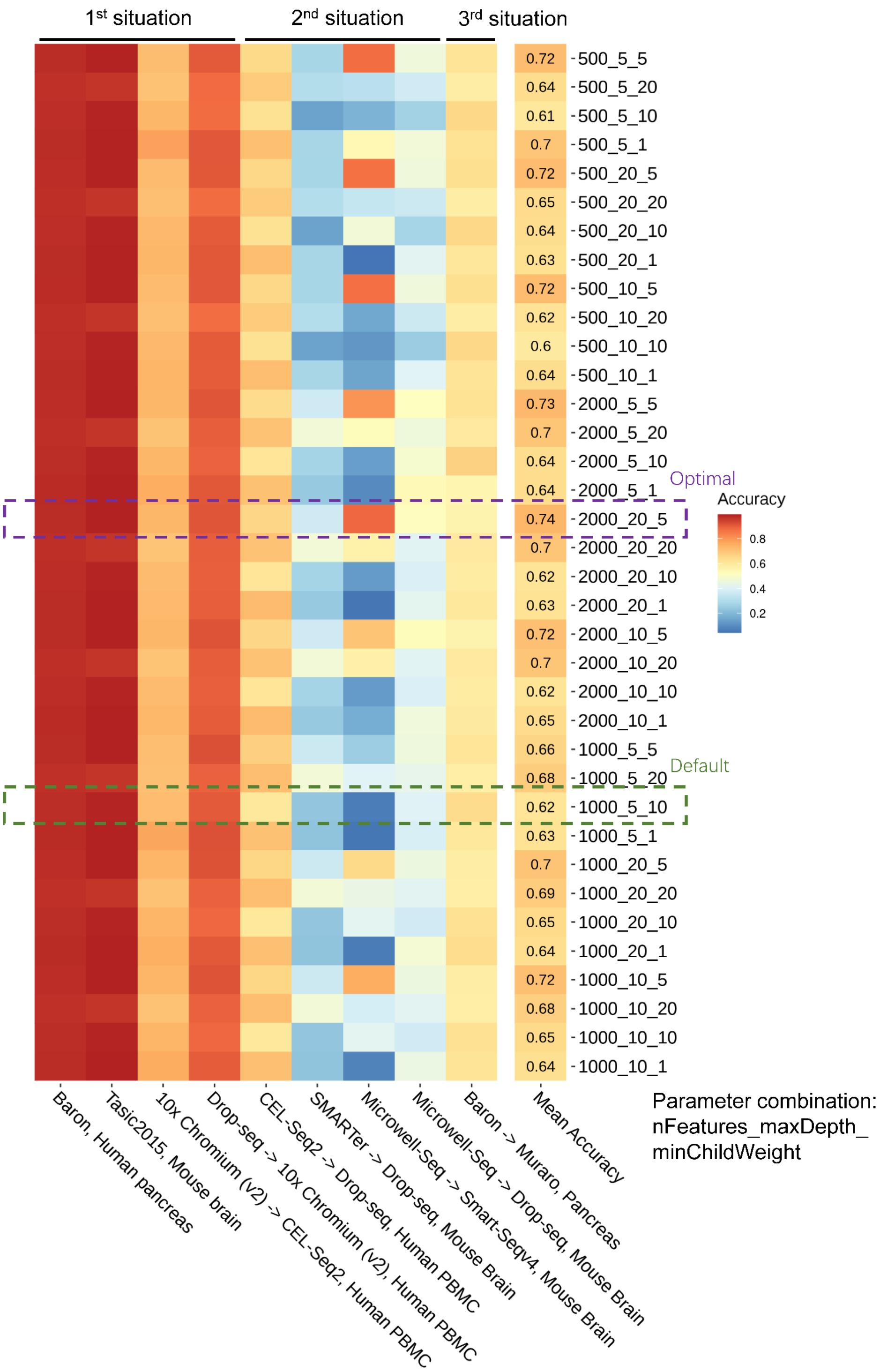

Supplementary Figure S13 The performance of CaSTLe in nine selected benchmark tests by using different combinations of parameters. There are three parameters to tune: “nFeatures” refers to the number of highly variable genes, “maxDepth” refers to the maximum depth of a tree, and “minChildWeight” refers to the minimum sum of instance weight needed in a child. Default and optimal parameter combination are highlighted by green and purple rectangle, respectively.

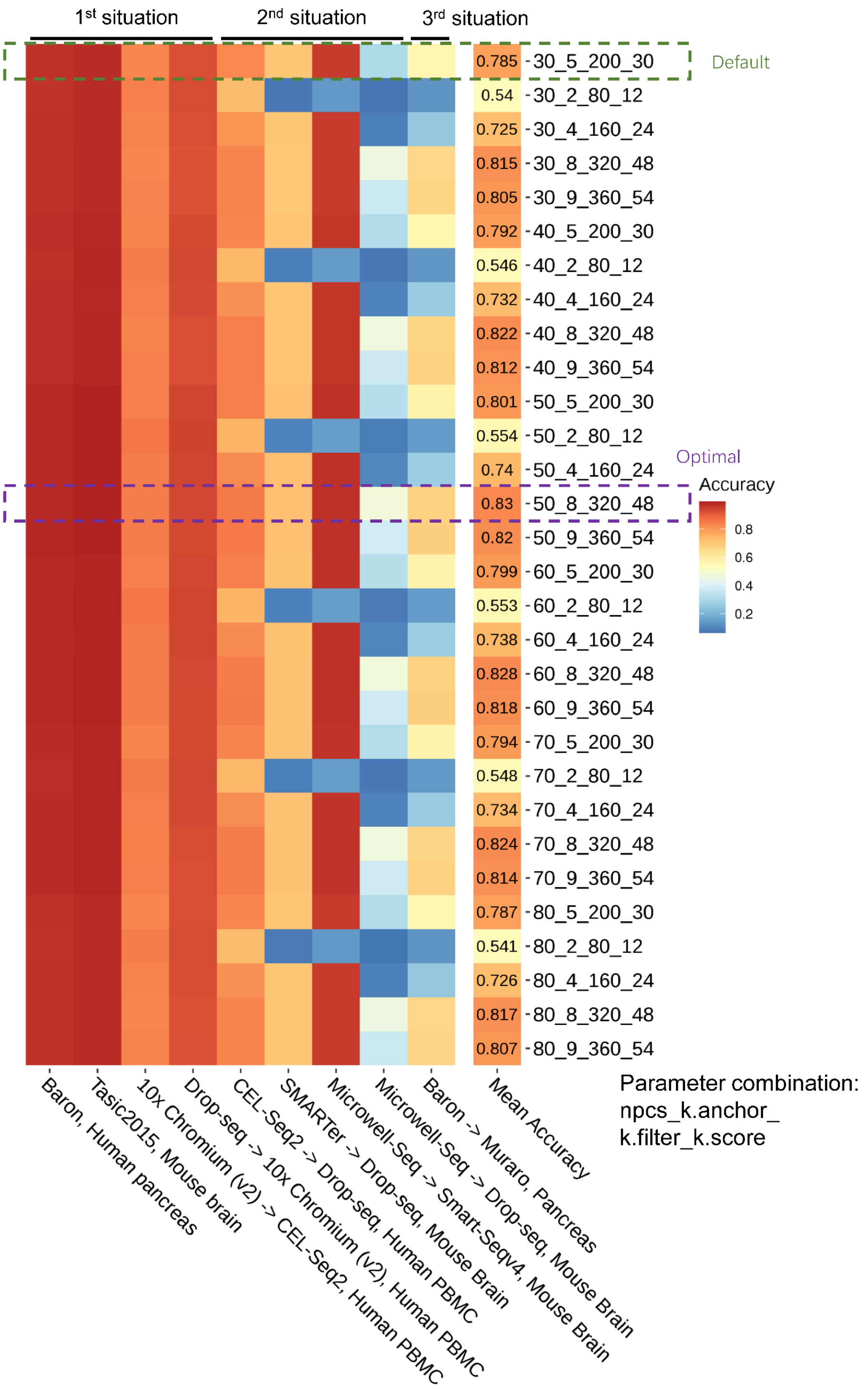

Supplementary Figure S14 The performance of Seurat v4 in nine selected benchmark tests by using different combinations of parameters. There are four parameters to tune: "npcs" refers to number of PCs used to specify the neighbor search space, "k.anchor", "k.filter", and "k.score" refers to neighbors used to find, filter, and score anchors defined as cell pairwise correspondences between single cells across datasets, respectively. Default and optimal parameter combination are highlighted by green and purple rectangle, respectively.

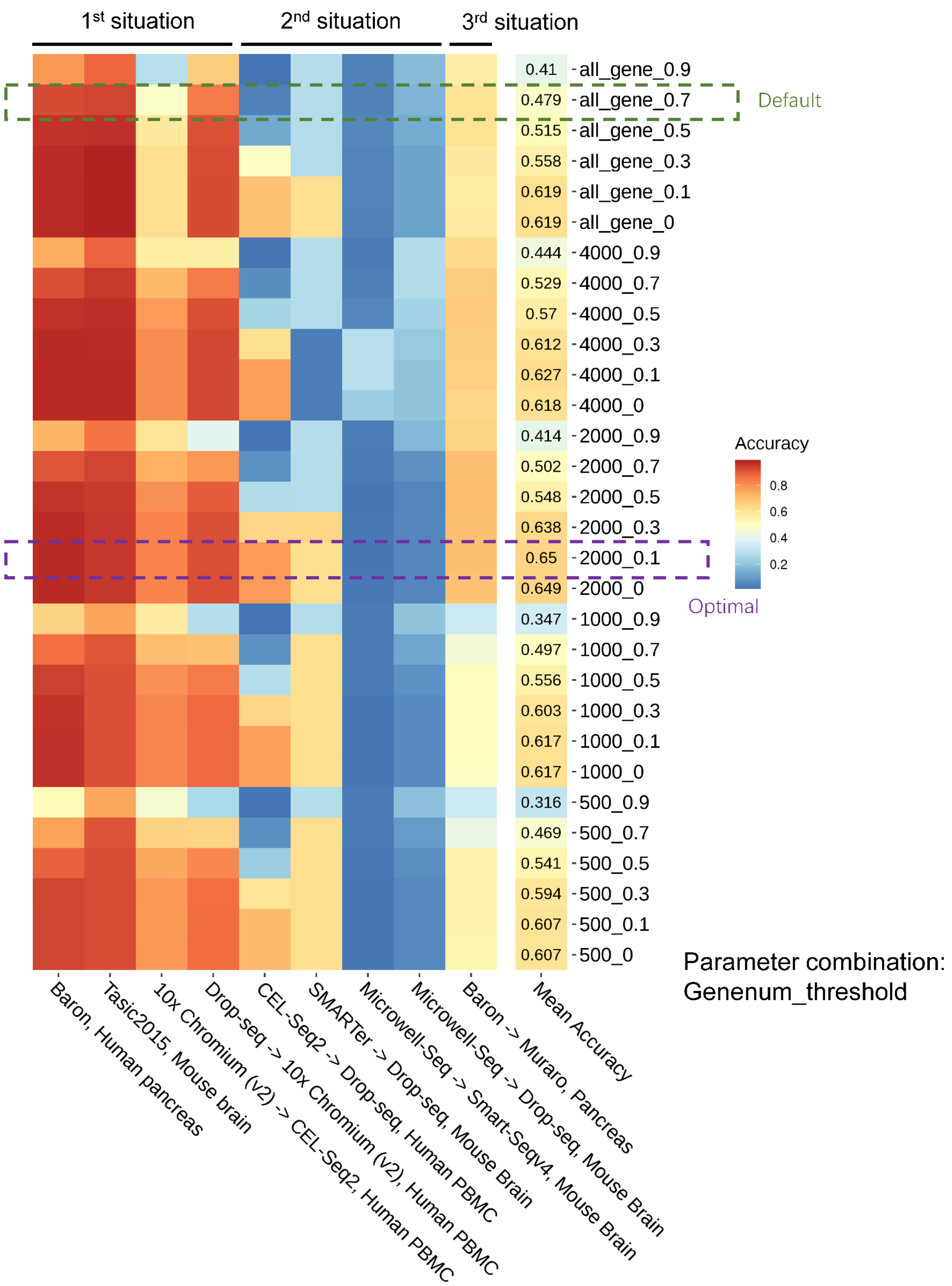

Supplementary Figure S15 The performance of SVM$_{rejection}$ in nine selected benchmark tests by using different combinations of parameters. There are two parameters to tune: "Genenum" refers to number of highly variable genes used to train the SVM model, "threshold" refers to the threshold of possibility values. Default and optimal parameter combination are highlighted by green and purple rectangle, respectively.

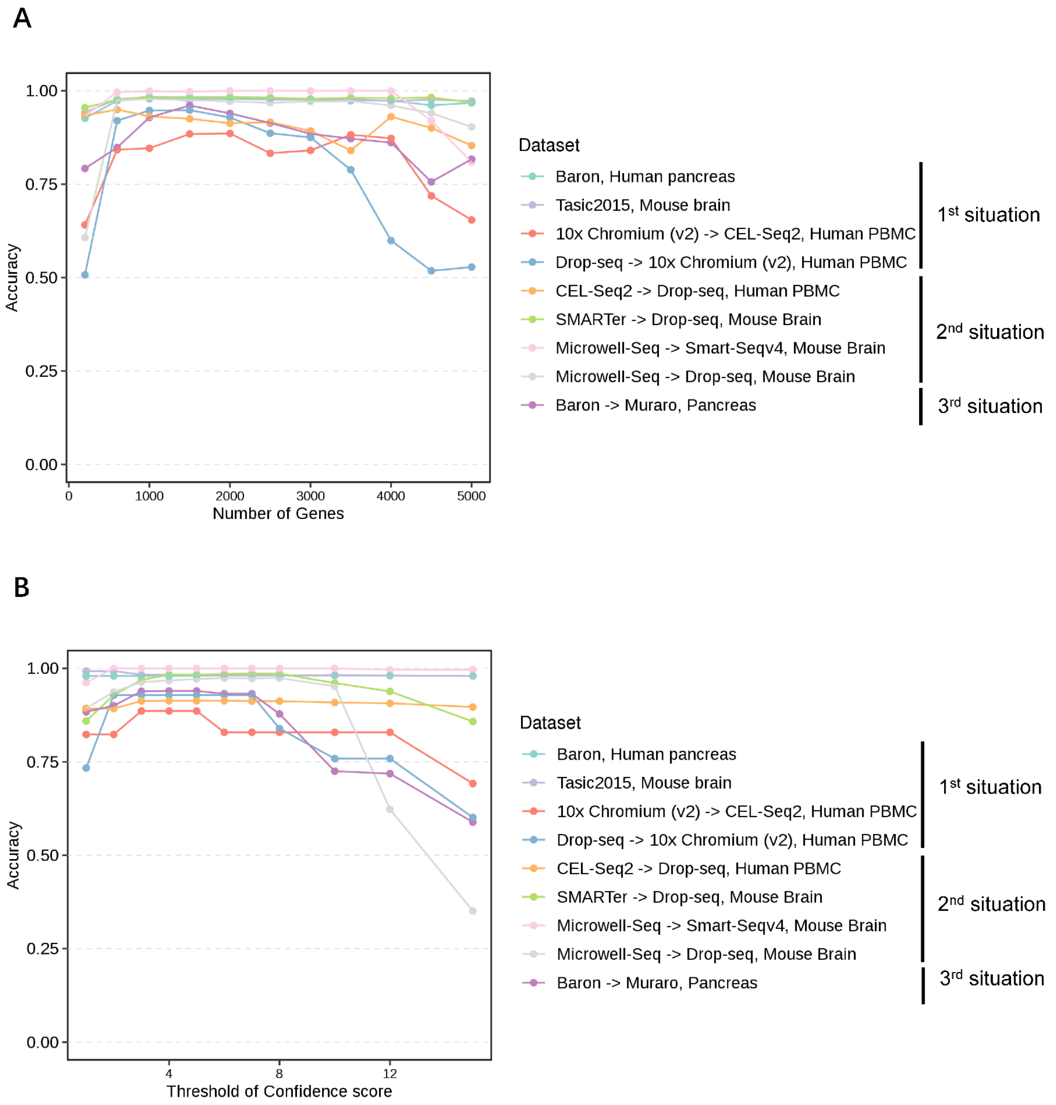

Supplementary Figure S16 Evaluation of the choice of parameters on the performance of scMAGIC (A) The accuracy of scMAGIC in the nine selected benchmark tests when different numbers of highly variable genes are used to calculate the similarity between the profiles of a query cell and a reference cell type. **(B)** is the same as in (A) except that different thresholds of confidence scores used to select query cells are tested.

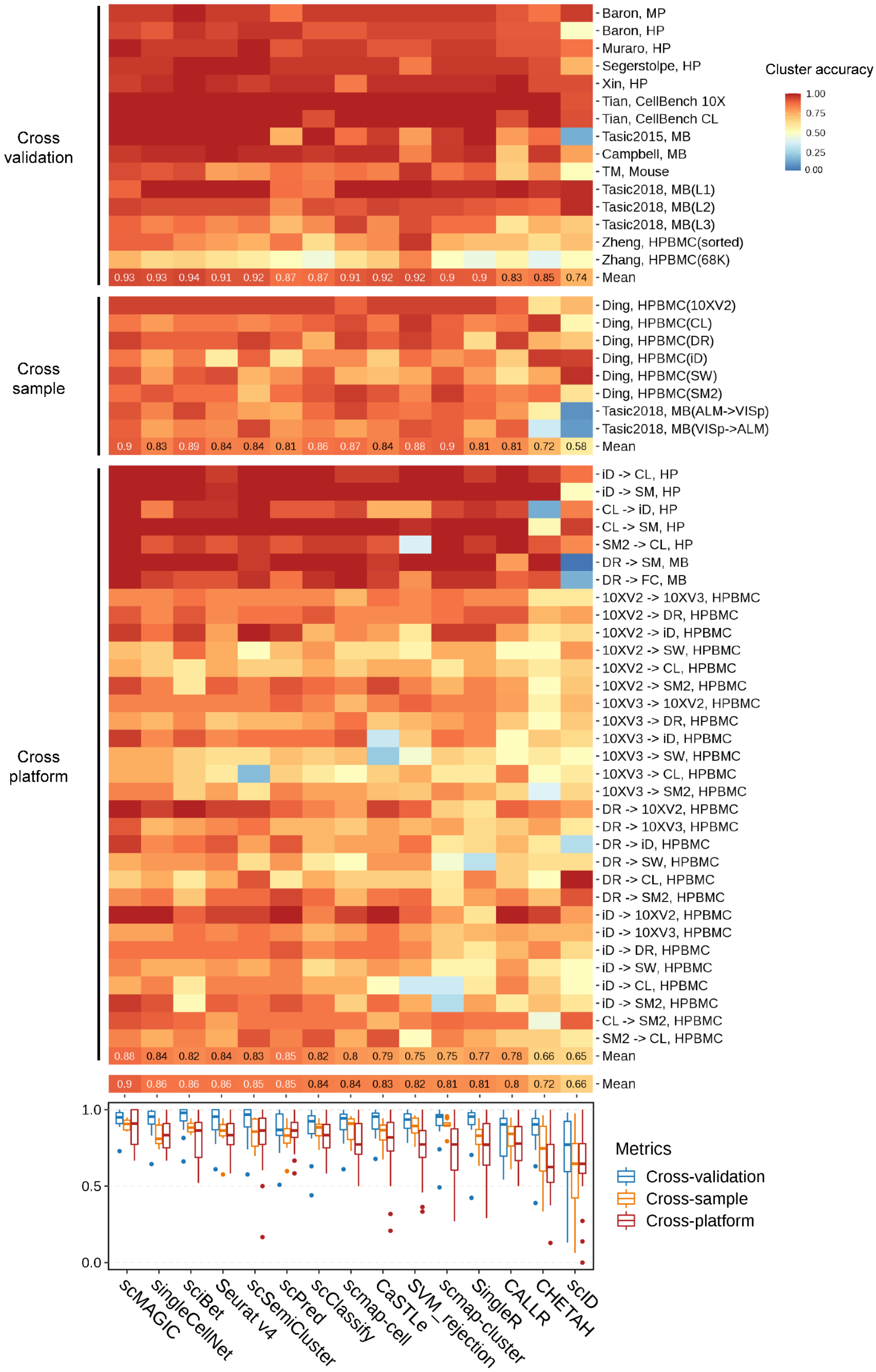

Supplementary Figure S17 The cluster accuracy of scMAGIC and 13 competing methods in the first situation. The figure is organized in the same way as **Figure 2** except that the benchmarking results of cluster accuracy are showed.

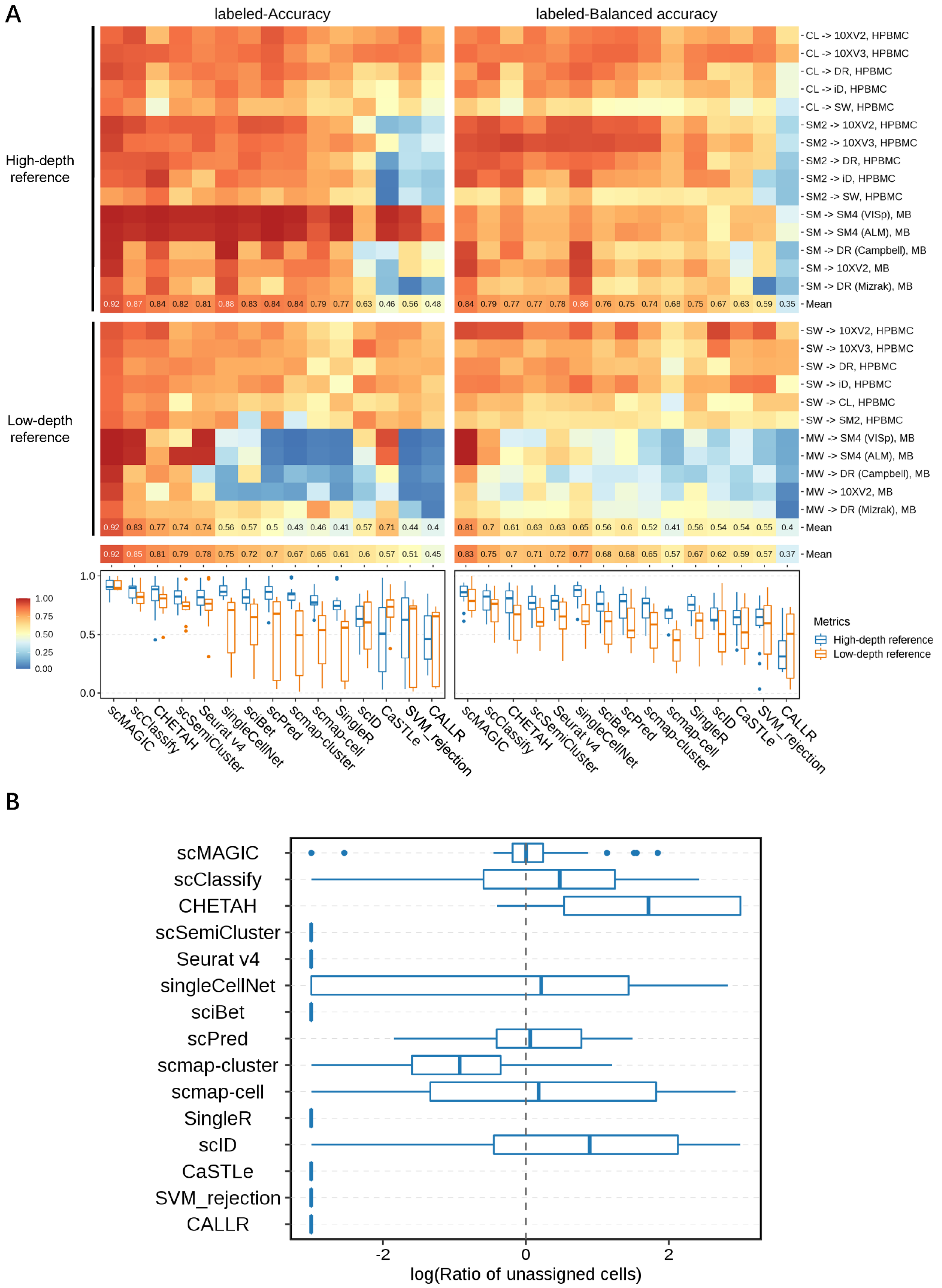

Supplementary Figure S18 The performance of scMAGIC and 13 competing methods in the second situation. (A) The figure is organized in the same way as **Figure 3A** except that the benchmarking results of labeled-accuracy and labeled-balanced accuracy are showed. **(B)** is the boxplot of the ratio of the number of query cells with "unassigned" labels to the number of unclassifiable query cells in natural logarithm of the 13 methods in the second situation. The closer the ratio to 0, the more accurate the method is for recognizing unclassifiable query cells.

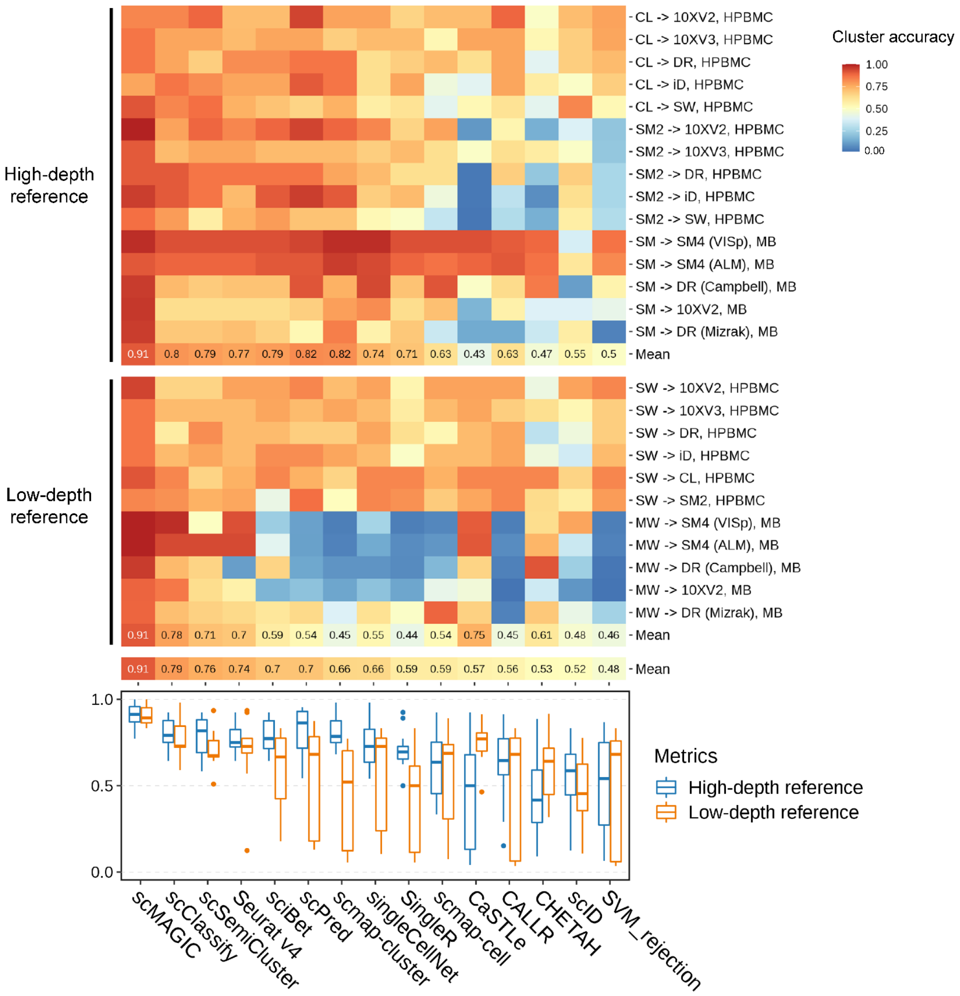

Supplementary Figure S19 The cluster accuracy of scMAGIC and 13 competing methods in the second situation. The figure is organized in the same way as **Figure 3A** except that the benchmarking results of cluster accuracy are showed.

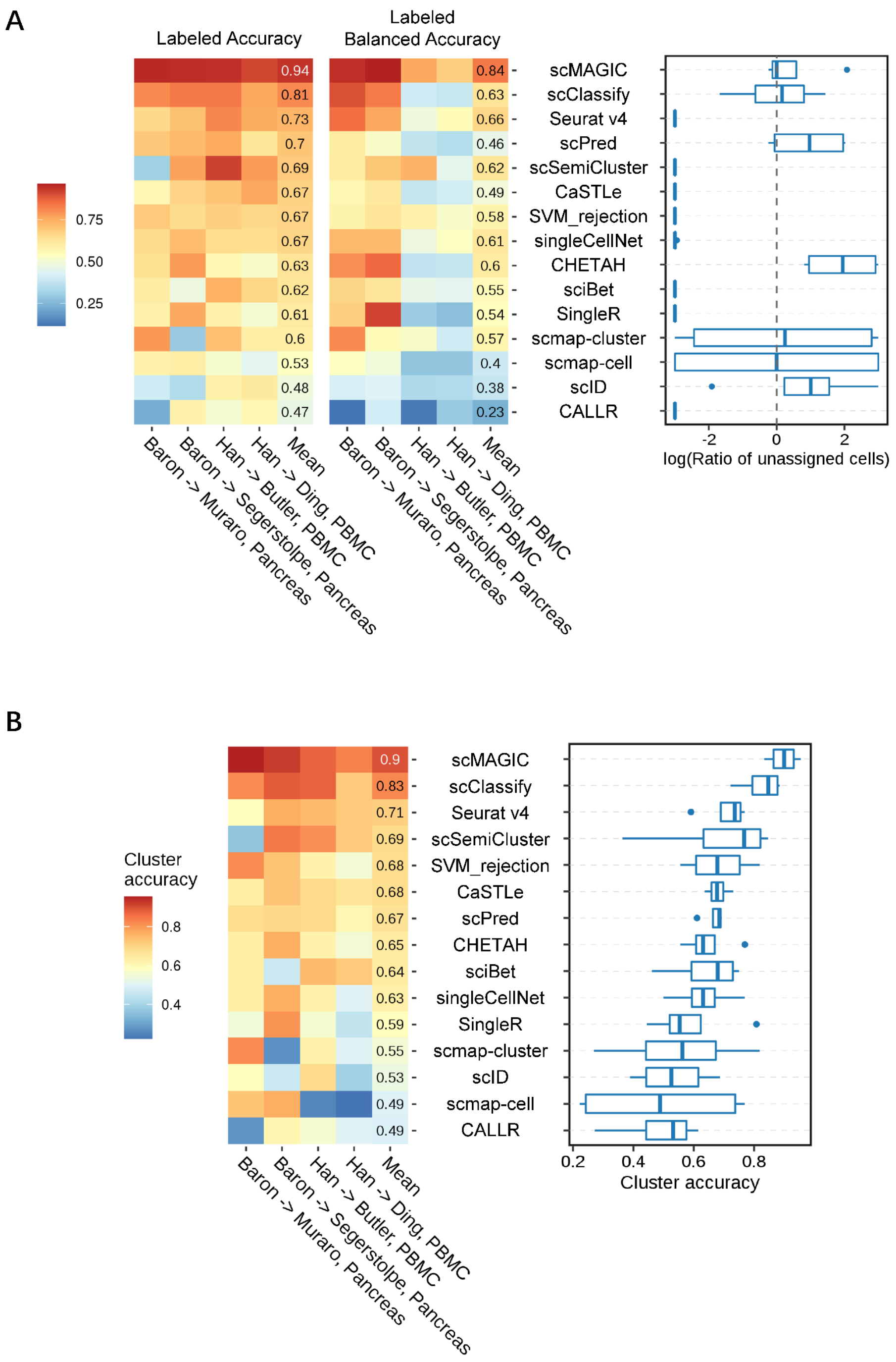

Supplementary Figure S20 The performance of scMAGIC and 13 competing methods in the third situation. (A) shows the heatmaps (left and middle) of the labeled accuracy and the labeled balanced accuracy of the 14 methods, and the boxplots (right) of the ratio of the number of query cells with "unassigned" labels to the number of unclassifiable query cells in natural logarithm of the 14 methods in the third situation. The 14 methods are arranged from top to bottom according to their mean labeled accuracy in decreasing order. **(B)** The cluster accuracy of 14 methods in the third situation is showed in heatmap (left) and boxplot (right). The 14 methods are arranged from top to bottom according to their mean cluster accuracy in decreasing order.

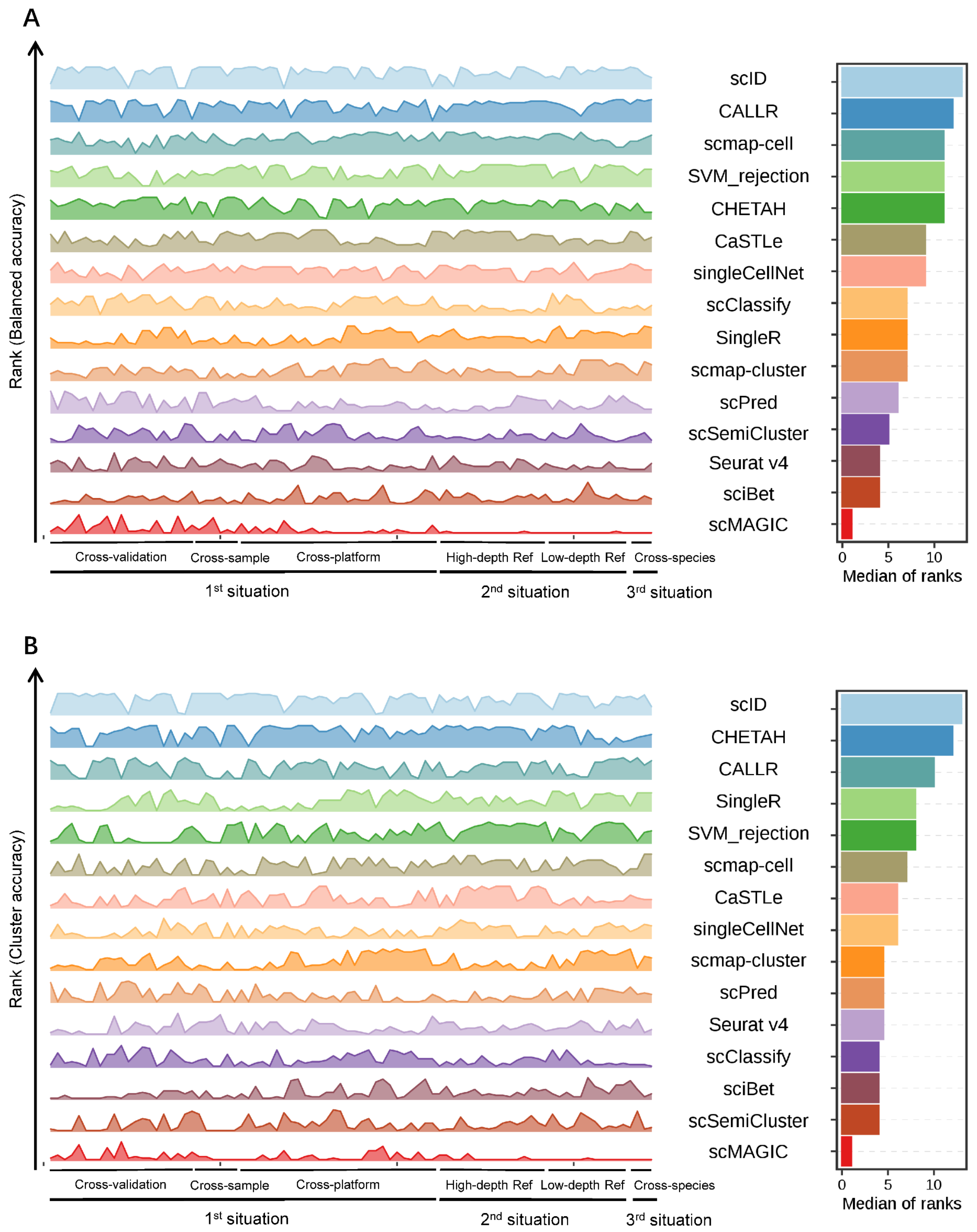

Supplementary Figure S21 The performance summary of scMAGIC and 13 competing methods across 86 benchmark tests. (A-B) shows the rank scores of the 14 methods ordered by their balanced accuracy **(A)** and cluster accuracy **(B)**, respectively, in each of the 86 benchmark tests corresponding to the three situations. The two plots are organized in the same way as **Figure 5A.**

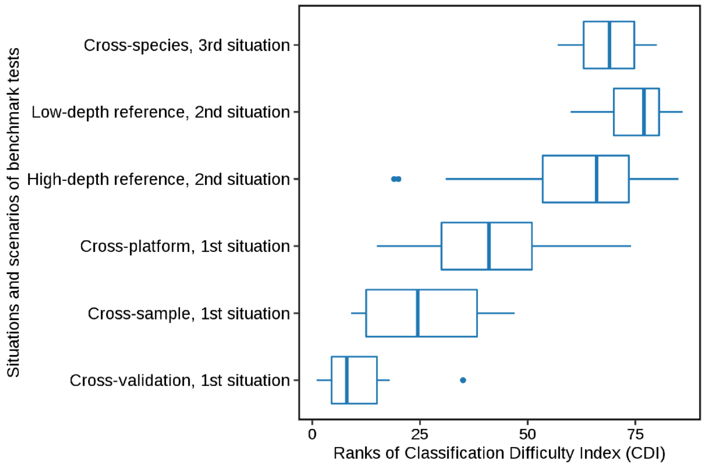

Supplementary Figure S22 The boxplot of the CDI ranks of benchmark tests belonging to different scenarios and situations.

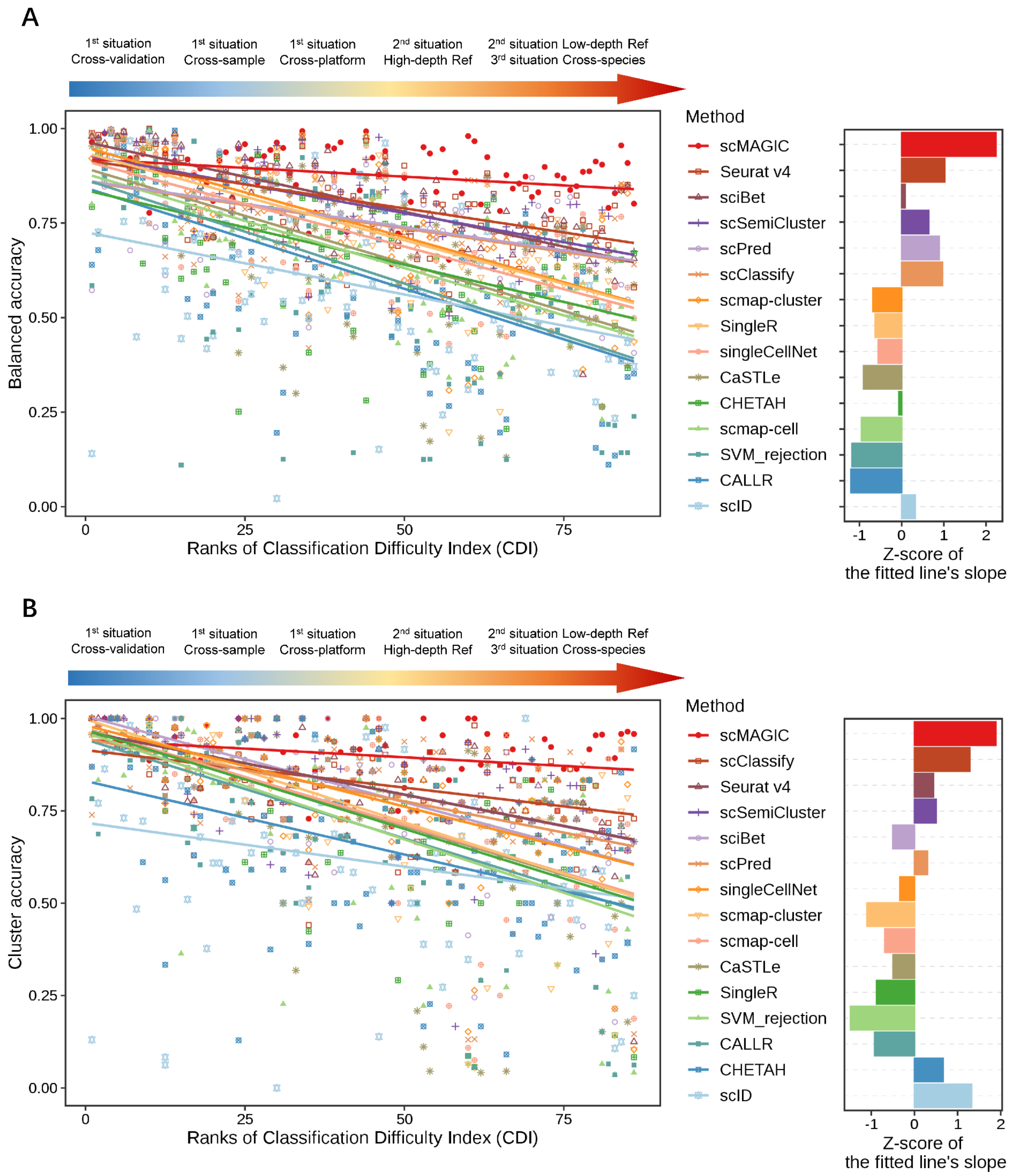

Supplementary Figure S23 The performance of scMAGIC and 13 competing methods with respect to the increase of classification difficulty. (A-B) are organized in the same way as **Figure 5A** expect that the accuracy is replaced with the balanced accuracy or cluster accuracy, respectively.

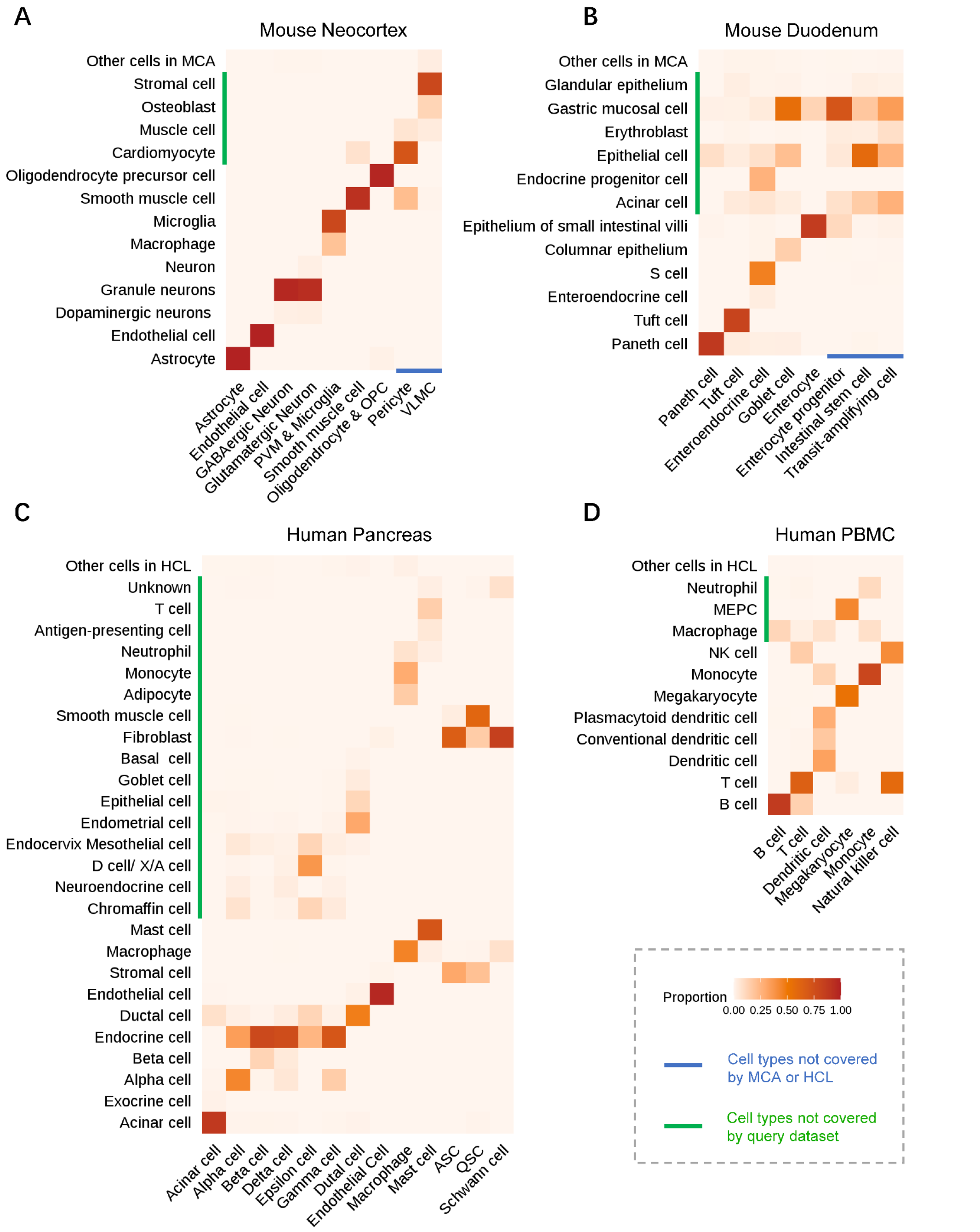

Supplementary Figure S24 The performance of scMCA and scHCL by using the atlas expression matrix as the reference. (**A-D**) show the heatmaps of the confusion matrix of scMCA or scHCL by using MCA (**A & B**) or HCL (**C & D**) as the reference to annotate mouse neocortex (B), mouse duodenum (**C**), human pancreas (**D**), and human PBMC, respectively. The four heatmaps are organized in the same way as those in **Figure 6**. PVM, peripheral vascular macrophage; OPC, oligodendrocyte precursor cell; VLMC, vascular and leptomeningeal cell; ASC, activated stellate cell; QSC, quiescent stellate cell; MEPC, Megakaryocyte/Erythtoid progenitor cell.

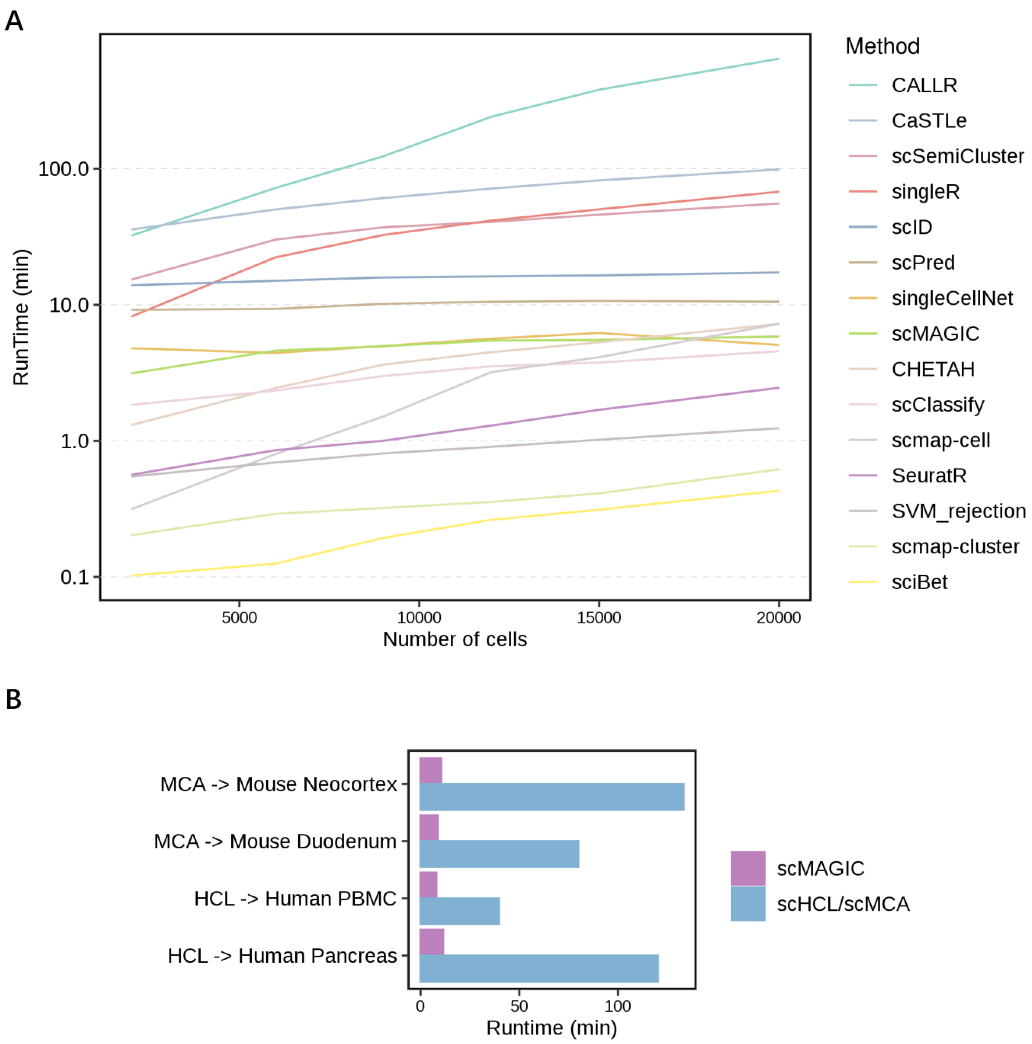

Supplementary Figure S25 Runtime evaluation of scMAGIC and 13 competing methods. (**A**) shows the runtimes of different methods by using the dataset of Campbell et al. downsampled with 2,000, 6,000, 9,000, 12,000, 15,000, and 20,000 cells as the query and the dataset of Hochgerner et al. as the reference. (**B**) shows the runtimes of scMAGIC, scMCA and scHCL in the four exploratory tasks.
